# Contributions to the knowledge of water bugs in Mindoro Island, Philippines, with a species checklist of Nepomorpha and Gerromorpha (Insecta, Hemiptera, Heteroptera)

**DOI:** 10.3897/BDJ.8.e56883

**Published:** 2020-11-04

**Authors:** Arthien Lovell Pelingen, Herbert Zettel, Clister V Pangantihon, Kyra Mari Dominique Aldaba, Earl Kevin Fatallo, Jemillie Madonna de Leon, Hendrik Freitag

**Affiliations:** 1 Ateneo de Manila University, Quezon City, Philippines Ateneo de Manila University Quezon City Philippines; 2 Natural History Museum, Vienna, Austria Natural History Museum Vienna Austria

**Keywords:** biodiversity, distribution, endemism, freshwater, macroinvertebrates

## Abstract

**Background:**

This survey aims to provide an updated species checklist of aquatic and semi-aquatic bugs in the intra-Philippine biogeographic Region of Mindoro. An assessment survey of water bugs (Hemiptera, Heteroptera) was conducted mostly by manual collection in selected areas of Oriental Mindoro from 2017 to 2018, in which some of the collecting activities were undertaken by graduate students of Ateneo de Manila University.

**New information:**

Twenty-nine aquatic and semi-aquatic heteropteran species were documented and some are known island-endemic species or subspecies, including *Enithares martini mindoroensis* Nieser & Zettel, 1999, *Hydrotrephes stereoides mindoroensis* Zettel, 2003, *Aphelocheirus freitagi* Zettel & Pangantihon, 2010, *Rhagovelia mindoroensis* Zettel, 1994, *Rhagovelia raddai* Zettel, 1994, *Rhagovelia potamophila* Zettel, 1996 and *Strongylovelia mindoroensis* Lansbury & Zettel, 1997, which were found in new areas in the Region. In addition, there are also new records for the Island that have already been documented in other parts of the Philippines, such as the Philippine-endemic *Ochterus magnus* Gapud & San Valentin, 1977 and *Hebrus philippinus* Zettel, 2006 and the widely-distributed backswimmers *Anisops nigrolineatus* Lundblad, 1933 and *Anisops rhomboides* Nieser & Chen, 1999. Several undescribed specimens and potentially new species are also discussed in this paper. Further surveys in the other parts of Mindoro and in the other regions of the Philippines, are encouraged to produce a comprehensive baseline data of heteropteran species richness in the country.

## Introduction

The order Hemiptera constitutes a large part of the insect fauna, both in terrestrial and aquatic ecosystems in the Philippines (Gapud 1986). The majority of the aquatic forms belong to Nepomorpha and Gerromorpha (water bugs) under the suborder Heteroptera (true bugs or typical bugs). These water bugs have been relatively well-studied compared to other aquatic macroinvertebrate taxa, mainly due to the comprehensive Philippine Water Bug Inventory Project (Gapud and Zettel 1999). Most of the represented species are Philippine-endemic, many of which are island-endemics. More than 200 species are known from the country, although numerous undescribed, or even undiscovered species still await their formal description. Despite their abundance, Heteroptera were subject to only a few studies in Mindoro. In Lake Naujan, Mindoro, 12 heteropteran species, along with 49 coleopteran species, were documented (Freitag and Pangantihon 2010), while in Lake Manguao Catchment, Palawan, 21 heteropteran species were recorded (Freitag and Zettel 2012). Zettel (2014) documented 85 gerromorphan species from the Island of Luzon, while Pangantihon and Freitag (2016) recorded 31 new island records of coastal and marine-associated heteropteran species from central Visayas and Mindoro. In total, 43 species were recorded from Mindoro in these previous studies. Since then, no published papers dealt with similar faunistic surveys on aquatic and semi-aquatic Heteroptera in any region of the Philippines. Thus, this study aims to update the species list of Gerromorpha and Nepomorpha of Mindoro. The majority of the survey efforts were accomplished within the scope of a graduate course activity by the junior authors in selected areas of the Island for which a Gratuitous Permit was issued by the Philippine Bureau of Fisheries and Aquatic Resources (BFAR).

Along with field collection from the easily-accessible municipalities of Puerto Galera and Baco, special attention is given to the Baroc River basin in Roxas, which belongs partly to the Key Biodiversity Area (KBA) “69 Hinunduang Mt.” with extremely high critical conservation priority (“EHc”) and high socioeconomic pressure (Ong et al. 2002). This study aims to address the lack of biodiversity data from Mindoro Island. The comprehensive assessment project of the Baroc River basin by the Ateneo Biodiversity Laboratory has led already to the discovery of several interesting aquatic arthropod species (Freitag 2013, Mey and Freitag 2013, Komarek and Freitag 2014, Vidal et al. 2017, Garces et al. 2018, Komarek and Freitag 2020, Pelingen and Freitag 2020). These papers also provide more details on some of the collecting sites.

## Materials and methods

### Abbreviations and acronyms

ap apterous

asl. above the sea level (elevation)

bra brachypterus

ma macropterous

N nymph

NN nymphs

s.l. sensu lato

s.str. sensu stricto

sp. species

ssp. subspecies

### Field Collection

A large proportion of the aquatic and semi-aquatic heteropteran material studied here was collected in rivers and streams of Oriental Mindoro from June 2017 to August 2018. The sampling sites (all from the Province of Oriental Mindoro, Philippines) are summarised in Table 1.

The main sampling method used was handpicking with the use of a hand-held net to collect the aquatic bugs. Some of the specimens had also been collected using a black light trap (L) or in emergence traps (E) as described by Freitag (2004).

In the label data of the material, the codes mentioned before for the collecting sites are followed by a single minor letter for the microhabitats (Fig. 3) listed and encoded in Table 2.

### Handling of Material Collected

Specimens collected were preserved in 96% ethanol and stored (-20°C) prior to identification. Morphological examination was done using a dissecting microscope (LEICA EZ4) and a compound microscope (OLYMPUS SZ61). The habitus images were produced using a Canon EOS 6D with macro lens and a stack rack. This stacking of images was operated using Helicon Remote and Helicon Focus. The figures were generated then processed with Adobe Photoshop.

Identification keys and other relevant literature were used for the taxa as stated in the respective taxonomic sections. In some cases, loaned type material from the Natural History Museum Vienna, Austria (NHMW) was used for comparison. The dissected parts and actual specimen were glued on to entomological papers, while some were stored in the vial with ethanol. All material is labelled and kept at the collections of the Biodiversity Laboratory, Ateneo de Manila University (ADMU), National Museum of the Philippines, Museum of Natural History, Manila, Philippines (NMP) and the Museum für Naturkunde Berlin, Germany (ZMB).

## Taxon treatments

### Ranatra sp.

9845DBEE-65C4-5E64-A78F-0EDE203B95D2

#### Materials

- **Type status:** Other material. **Occurrence:** individualCount: 2 males (ma); **Taxon:** scientificName: *Ranatra* sp.; **Location:** island: Mindoro; country: Philippines; municipality: Puerto Galera; locationRemarks: 305g; **Event:** eventDate: 22.06.2017
- **Type status:** Other material. **Occurrence:** individualCount: 1 female (ma); **Taxon:** scientificName: *Ranatra* sp.; **Location:** island: Mindoro; country: Philippines; municipality: Baco; locationRemarks: 353e; **Event:** eventDate: 24.08.2017

#### Taxon discussion

This still unnamed new species (Fig. 4A) of the *Ranatra gracilis* Dallas, 1850 group (see Dallas 1850, Lansbury 1972) will be described and discussed further in an upcoming paper by Tran and Zettel (in prep.).

#### Habitat

**Figure 1.**
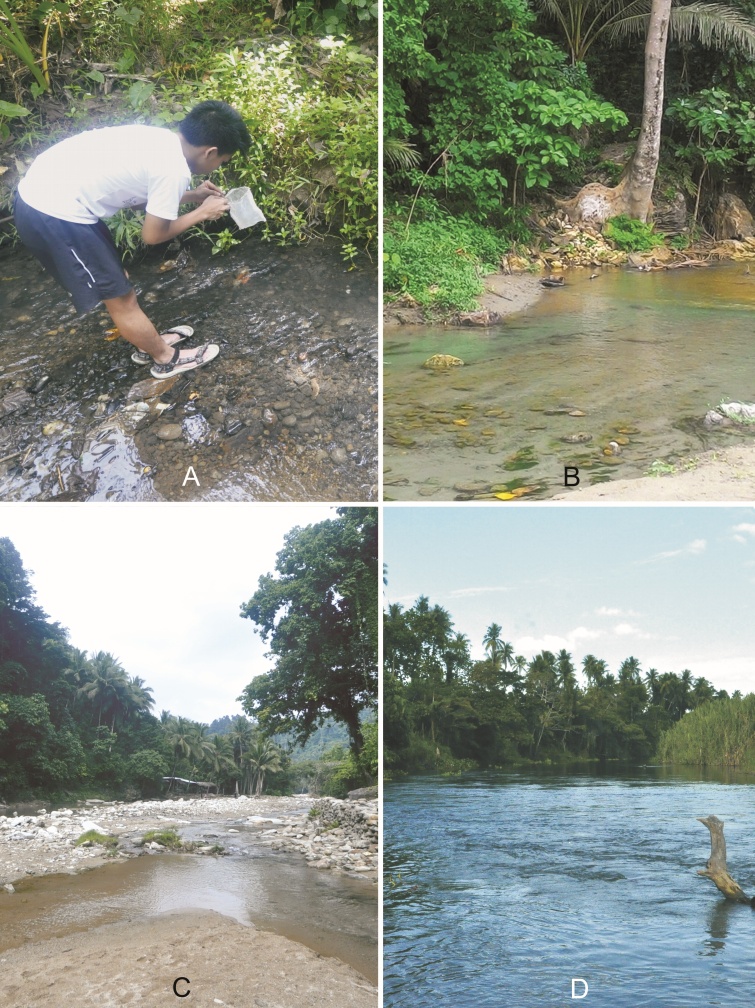
Sampling sites at northern Mindoro (Baco, Puerto Galera). **A.** the first author sampling at site 396; **B.** Tagbinai Malaki River (305); **C.** Tukunan River at "Hidden Paradise" (303); **D.** lower Dulangan River (356).

Specimens were found in both flowing and calm littoral sections of shallow streams, such as in Fig. 1B. See Fig. 5 for current records.

### Ochterus magnus

Gapud & San Valentin, 1977

C8C613DA-E50D-5E82-89CC-852C48F824D1

#### Materials

- **Type status:** Other material. **Occurrence:** individualCount: 3 males (ma); **Taxon:** scientificName: Ochterus magnus; **Location:** island: Mindoro; country: Philippines; municipality: Roxas; locationRemarks: TIRj; **Event:** eventDate: 05.07.2017

#### Distribution

This is the first record of *O. magnus* Gapud & San Valentin, 1977 (see Gapud and San Valentin 1977) from Mindoro (Fig. 5). The species is Philippine-endemic, previously only known from Luzon – Mt. Makiling, Laguna; Humayao Creek, Cavite; Quezon; and in La Union (Gapud 2002).

#### Taxon discussion

Refer to Gapud (1986) for identification.

#### Habitat

The specimens were found on wet rocks.

### Ochterus polhemusi

Gapud, 1981

F36778EB-E735-5BA6-B409-0E2F2EF63C9C

#### Materials

- **Type status:** Other material. **Occurrence:** individualCount: 1 female (ma); **Taxon:** scientificName: Ochterus polhemusi; **Location:** island: Mindoro; country: Philippines; municipality: Roxas; locationRemarks: HBT(E); **Event:** eventDate: 21.09.2017
- **Type status:** Other material. **Occurrence:** individualCount: 3 males (ma); **Taxon:** scientificName: Ochterus polhemusi; **Location:** island: Mindoro; country: Philippines; municipality: Roxas; locationRemarks: HQCk; **Event:** eventDate: 21.09.2017
- **Type status:** Other material. **Occurrence:** individualCount: 1 female (ma); **Taxon:** scientificName: Ochterus polhemusi; **Location:** island: Mindoro; country: Philippines; municipality: Roxas; locationRemarks: HQC(E); **Event:** eventDate: 28.09.2017
- **Type status:** Other material. **Occurrence:** individualCount: 4 males (ma); **Taxon:** scientificName: Ochterus polhemusi; **Location:** island: Mindoro; country: Philippines; municipality: Roxas; locationRemarks: TWCj; **Event:** eventDate: 23.09.2017

#### Distribution

This species (Fig. 4B) is widely distributed in the Philippines. See Fig. 5 for our additional records.

#### Taxon discussion

Refer to Gapud (1981) for identification. In Mindoro, three species of *Ochterus* Latreille, 1807 are recorded so far, O. marginatus (Latreille, 1804) ssp. insularis Rieger, 1977, *O. polhemusi* Gapud, 1981 and *O. philippinensis* Kormilev, 1971 (Kormilev 1971, Gapud 1995, Gapud 2002).

#### Habitat

We collected specimens in emergence traps spanned over littoral portions of small streams, on hygropetric rocks and along small rivulets. The species is generally found in banks of streams, ponds, lakes, freshwater marshlands and in association with waterfalls. It is also found in substrates that are muddy or sandy.

### Micronecta sp.

6CD92895-F3EB-5B65-A5D4-9E3B0674434B

#### Materials

- **Type status:** Other material. **Occurrence:** individualCount: 18 males (ma); **Taxon:** scientificName: *Micronecta* sp.; **Location:** island: Mindoro; country: Philippines; municipality: Roxas; locationRemarks: HR2(L); **Event:** eventDate: 31.06.2017
- **Type status:** Other material. **Occurrence:** individualCount: 4 males (ma); **Taxon:** scientificName: *Micronecta* sp.; **Location:** island: Mindoro; country: Philippines; municipality: Roxas; locationRemarks: TR1(L); **Event:** eventDate: 13.07.2017
- **Type status:** Other material. **Occurrence:** individualCount: 2 males (ma); **Taxon:** scientificName: *Micronecta* sp.; **Location:** island: Mindoro; country: Philippines; municipality: Roxas; locationRemarks: TR2(L); **Event:** eventDate: 21.06.2017
- **Type status:** Other material. **Occurrence:** individualCount: 1 female (ma); **Taxon:** scientificName: *Micronecta* sp.; **Location:** island: Mindoro; country: Philippines; municipality: Roxas; locationRemarks: TR2(L); **Event:** eventDate: 20.08.2018

#### Taxon discussion

The species cannot be identified yet because the examination of comparative type material is needed, which is currently inaccessible. Nieser and Chen (2003) described four species from the Philippines but the Mindoro specimens differ from those.

#### Habitat

**Figure 2.**
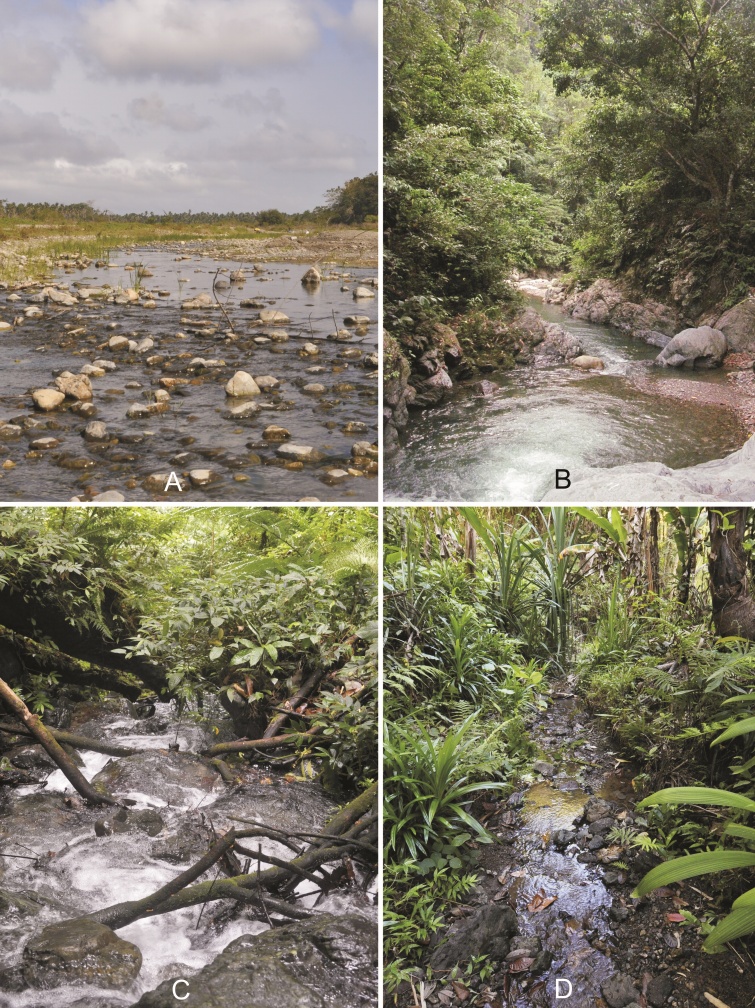
Sampling sites at the Baroc River basin (Roxas, southern Mindoro). **A.** lower Baroc River (BR3); **B.** Hinundungan River from Hinagdanan Falls (HR2); **C.** upper Taugad Daka River (TDR4); **D.** small tributary of the Taugad River (TUC).

All specimens were collected using a light trap, so their habitat cannot be accurately described. However, species of *Micronecta* Kirkaldy, 1897 are usually found in stagnant or shallow, slowly flowing waters (Nieser 1999). See Fig. 5 for current records, amongst them is site HR2 (Fig. 2B).

### Asthenocoris luzonensis paradisianus

Zettel & Nieser, 1999

EAC5BABD-8DB0-57BC-BB7C-B9661DE764AC

#### Materials

- **Type status:** Other material. **Occurrence:** individualCount: 1 female (bra); **Taxon:** scientificName: Asthenocoris luzonensis paradisianus; **Location:** island: Mindoro; country: Philippines; municipality: Puerto Galera; locationRemarks: 303b2; **Event:** eventDate: 06/07/2017
- **Type status:** Other material. **Occurrence:** individualCount: 1 female (bra); **Taxon:** scientificName: Asthenocoris luzonensis paradisianus; **Location:** island: Mindoro; country: Philippines; municipality: Baco; locationRemarks: 310c; **Event:** eventDate: 08/22/2017
- **Type status:** Other material. **Occurrence:** individualCount: 1 female (bra); **Taxon:** scientificName: Asthenocoris luzonensis paradisianus; **Location:** island: Mindoro; country: Philippines; municipality: Baco; locationRemarks: 312f1; **Event:** eventDate: 08/22/2017
- **Type status:** Other material. **Occurrence:** individualCount: 1 female (bra); **Taxon:** scientificName: Asthenocoris luzonensis paradisianus; **Location:** island: Mindoro; country: Philippines; municipality: Roxas; locationRemarks: HBCa; **Event:** eventDate: 06/20/2017
- **Type status:** Other material. **Occurrence:** individualCount: 1 female (bra); **Taxon:** scientificName: Asthenocoris luzonensis paradisianus; **Location:** island: Mindoro; country: Philippines; municipality: Roxas; locationRemarks: HBTc; **Event:** eventDate: 07/05/2017
- **Type status:** Other material. **Occurrence:** individualCount: 1 female (bra); **Taxon:** scientificName: Asthenocoris luzonensis paradisianus; **Location:** island: Mindoro; country: Philippines; municipality: Roxas; locationRemarks: HQCc; **Event:** eventDate: 06/16/2017
- **Type status:** Other material. **Occurrence:** individualCount: 18 males (bra), 1 female (bra); **Taxon:** scientificName: Asthenocoris luzonensis paradisianus; **Location:** island: Mindoro; country: Philippines; municipality: Roxas; locationRemarks: HQCc; **Event:** eventDate: 06/30/2017
- **Type status:** Other material. **Occurrence:** individualCount: 2 males (bra); **Taxon:** scientificName: Asthenocoris luzonensis paradisianus; **Location:** island: Mindoro; country: Philippines; municipality: Roxas; locationRemarks: HQCh; **Event:** eventDate: 09/26/2017
- **Type status:** Other material. **Occurrence:** individualCount: 3 males (bra); **Taxon:** scientificName: Asthenocoris luzonensis paradisianus; **Location:** island: Mindoro; country: Philippines; municipality: Roxas; locationRemarks: HR1c; **Event:** eventDate: 06/17/2017
- **Type status:** Other material. **Occurrence:** individualCount: 5 males (bra), 1 female (bra); **Taxon:** scientificName: Asthenocoris luzonensis paradisianus; **Location:** island: Mindoro; country: Philippines; municipality: Roxas; locationRemarks: HR1c; **Event:** eventDate: 07/08/2017
- **Type status:** Other material. **Occurrence:** individualCount: 2 males (bra); **Taxon:** scientificName: Asthenocoris luzonensis paradisianus; **Location:** island: Mindoro; country: Philippines; municipality: Roxas; locationRemarks: HR2c; **Event:** eventDate: 07/03/2017
- **Type status:** Other material. **Occurrence:** individualCount: 3 males (bra); **Taxon:** scientificName: Asthenocoris luzonensis paradisianus; **Location:** island: Mindoro; country: Philippines; municipality: Roxas; locationRemarks: HR2d; **Event:** eventDate: 12/06/2017
- **Type status:** Other material. **Occurrence:** individualCount: 1 female (bra); **Taxon:** scientificName: Asthenocoris luzonensis paradisianus; **Location:** island: Mindoro; country: Philippines; municipality: Roxas; locationRemarks: HR3c; **Event:** eventDate: 31.06.2017
- **Type status:** Other material. **Occurrence:** individualCount: 1 female (bra); **Taxon:** scientificName: Asthenocoris luzonensis paradisianus; **Location:** island: Mindoro; country: Philippines; municipality: Roxas; locationRemarks: HR3c; **Event:** eventDate: 03/31/2018
- **Type status:** Other material. **Occurrence:** individualCount: 1 female (bra); **Taxon:** scientificName: Asthenocoris luzonensis paradisianus; **Location:** island: Mindoro; country: Philippines; municipality: Roxas; locationRemarks: HTCc; **Event:** eventDate: 07/03/2017
- **Type status:** Other material. **Occurrence:** individualCount: 1 female (bra); **Taxon:** scientificName: Asthenocoris luzonensis paradisianus; **Location:** island: Mindoro; country: Philippines; municipality: Roxas; locationRemarks: HTCf; **Event:** eventDate: 12/28/2017
- **Type status:** Other material. **Occurrence:** individualCount: 2 males (bra), 1 female (bra); **Taxon:** scientificName: Asthenocoris luzonensis paradisianus; **Location:** island: Mindoro; country: Philippines; municipality: Roxas; locationRemarks: TDR1c; **Event:** eventDate: 06/18/2017
- **Type status:** Other material. **Occurrence:** individualCount: 1 female (bra); **Taxon:** scientificName: Asthenocoris luzonensis paradisianus; **Location:** island: Mindoro; country: Philippines; municipality: Roxas; locationRemarks: TDR1f; **Event:** eventDate: 07/08/2017
- **Type status:** Other material. **Occurrence:** individualCount: 1 female (bra); **Taxon:** scientificName: Asthenocoris luzonensis paradisianus; **Location:** island: Mindoro; country: Philippines; municipality: Roxas; locationRemarks: TDR1f; **Event:** eventDate: 06/18/2017
- **Type status:** Other material. **Occurrence:** individualCount: 1 female (bra); **Taxon:** scientificName: Asthenocoris luzonensis paradisianus; **Location:** island: Mindoro; country: Philippines; municipality: Roxas; locationRemarks: TDR1f; **Event:** eventDate: 09/22/2017
- **Type status:** Other material. **Occurrence:** individualCount: 1 female (bra); **Taxon:** scientificName: Asthenocoris luzonensis paradisianus; **Location:** island: Mindoro; country: Philippines; municipality: Roxas; locationRemarks: TDR1c; **Event:** eventDate: 04/04/2018
- **Type status:** Other material. **Occurrence:** individualCount: 1 female (bra); **Taxon:** scientificName: Asthenocoris luzonensis paradisianus; **Location:** island: Mindoro; country: Philippines; municipality: Roxas; locationRemarks: THCf; **Event:** eventDate: 06/23/2017
- **Type status:** Other material. **Occurrence:** individualCount: 2 males (bra); **Taxon:** scientificName: Asthenocoris luzonensis paradisianus; **Location:** island: Mindoro; country: Philippines; municipality: Roxas; locationRemarks: THCd; **Event:** eventDate: 11/17/2017
- **Type status:** Other material. **Occurrence:** individualCount: 1 female (bra); **Taxon:** scientificName: Asthenocoris luzonensis paradisianus; **Location:** island: Mindoro; country: Philippines; municipality: Roxas; locationRemarks: TIR1d; **Event:** eventDate: 12/05/2017
- **Type status:** Other material. **Occurrence:** individualCount: 2 males (bra); **Taxon:** scientificName: Asthenocoris luzonensis paradisianus; **Location:** island: Mindoro; country: Philippines; municipality: Roxas; locationRemarks: TIR1d; **Event:** eventDate: 09/22/2017
- **Type status:** Other material. **Occurrence:** individualCount: 2 males (bra), 1 female (bra); **Taxon:** scientificName: Asthenocoris luzonensis paradisianus; **Location:** island: Mindoro; country: Philippines; municipality: Roxas; locationRemarks: TR1h; **Event:** eventDate: 07/02/2017
- **Type status:** Other material. **Occurrence:** individualCount: 1 female (bra); **Taxon:** scientificName: Asthenocoris luzonensis paradisianus; **Location:** island: Mindoro; country: Philippines; municipality: Roxas; locationRemarks: TR1d; **Event:** eventDate: 09/22/2017
- **Type status:** Other material. **Occurrence:** individualCount: 3 males (bra); **Taxon:** scientificName: Asthenocoris luzonensis paradisianus; **Location:** island: Mindoro; country: Philippines; municipality: Roxas; locationRemarks: TR2f; **Event:** eventDate: 06/14/2017
- **Type status:** Other material. **Occurrence:** individualCount: 3 males (bra); **Taxon:** scientificName: Asthenocoris luzonensis paradisianus; **Location:** island: Mindoro; country: Philippines; municipality: Roxas; locationRemarks: TR2c; **Event:** eventDate: 07/03/2017
- **Type status:** Other material. **Occurrence:** individualCount: 2 males (bra), 1 female (bra); **Taxon:** scientificName: Asthenocoris luzonensis paradisianus; **Location:** island: Mindoro; country: Philippines; municipality: Roxas; locationRemarks: TR2d; **Event:** eventDate: 07/07/2017
- **Type status:** Other material. **Occurrence:** individualCount: 2 males (bra); **Taxon:** scientificName: Asthenocoris luzonensis paradisianus; **Location:** island: Mindoro; country: Philippines; municipality: Roxas; locationRemarks: TR2h; **Event:** eventDate: 11/17/2017

#### Distribution

This subspecies is endemic to Mindoro (Zettel et al. 1999). See Fig. 5 for new records.

#### Taxon discussion

For identification, refer to the key by Zettel et al. (1999), a habitus illustration is provided in Fig. 4C. The genus *Asthenocoris* is endemic to the Philippines (Zettel et al. 1999). All material treated in here is brachypterous.

#### Habitat

**Figure 3.**
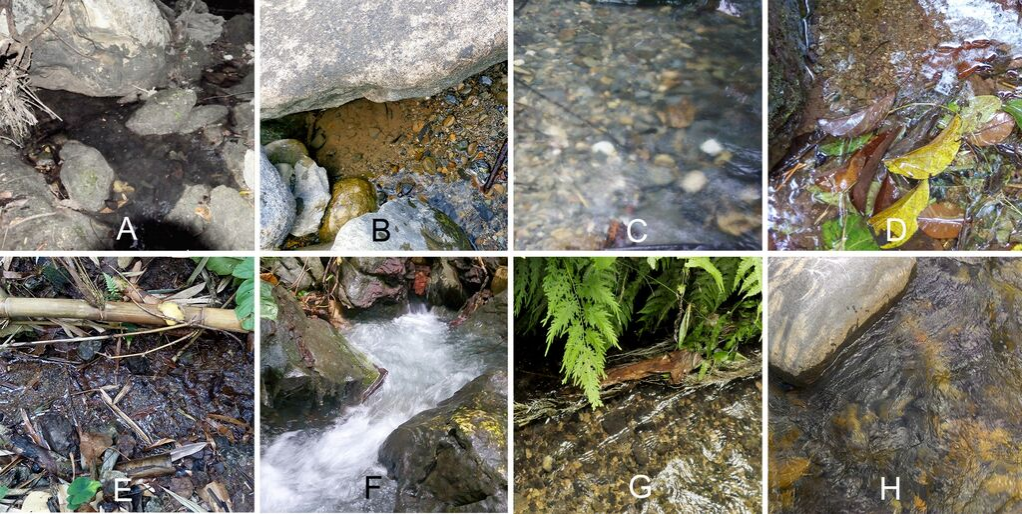
Microhabitats sampled with their respective label codes, as listed above. **A.** side pool with mineral deposits (“t”); **B.** littoral pool with mineral deposits (“b”); **C.** bottom gravel in running water (“c”); **D.** leaf packs trapped in riffles (“d”); **E.** leaf litter in rivulet (“k”); **F.** rock surface in riffle (“g”); **G.** root packs in running water (“h”); **H.** neustic in running water (“z”).

*Asthenocoris luzonensis paradisianus* Zetttel & Nieser, 2009 Fig. 4C is found in middle-sized streams running through secondary rainforest (Fig. 2B), as well as large, fast flowing streams, partly in secondary vegetation (Fig. 1C), but then only downstream of forested areas (Zettel et al. 1999). The specimens were retrieved in more or less fast flowing water from several substrates, foremost gravel, but also wood, leaf litter and root packs (Fig. 3G).

### Aphelocheirus freitagi

Zettel & Pangantihon, 2010

3FCEDFAD-987F-538B-AAEF-186925E7E5F6

#### Materials

- **Type status:** Other material. **Occurrence:** individualCount: 1 (N); **Taxon:** scientificName: Aphelocheirus freitagi; **Location:** island: Mindoro; country: Philippines; municipality: Baco; locationRemarks: 356c; **Event:** eventDate: 29.08.2017
- **Type status:** Other material. **Occurrence:** individualCount: 2 (NN); **Taxon:** scientificName: Aphelocheirus freitagi; **Location:** island: Mindoro; country: Philippines; municipality: Roxas; locationRemarks: BR3m; **Event:** eventDate: 04.12.2017
- **Type status:** Other material. **Occurrence:** individualCount: 7 males (bra); **Taxon:** scientificName: Aphelocheirus freitagi; **Location:** island: Mindoro; country: Philippines; municipality: Roxas; locationRemarks: BR3b; **Event:** eventDate: 22.07.2017
- **Type status:** Other material. **Occurrence:** individualCount: 4 males (bra); **Taxon:** scientificName: Aphelocheirus freitagi; **Location:** island: Mindoro; country: Philippines; municipality: Roxas; locationRemarks: HR1e; **Event:** eventDate: 17.06.2017
- **Type status:** Other material. **Occurrence:** individualCount: 1 female (bra); **Taxon:** scientificName: Aphelocheirus freitagi; **Location:** island: Mindoro; country: Philippines; municipality: Roxas; locationRemarks: HR2e; **Event:** eventDate: 03.07.2017
- **Type status:** Other material. **Occurrence:** individualCount: 1 female (bra); **Taxon:** scientificName: Aphelocheirus freitagi; **Location:** island: Mindoro; country: Philippines; municipality: Roxas; locationRemarks: TDR1c; **Event:** eventDate: 18.06.2017
- **Type status:** Other material. **Occurrence:** individualCount: 1 (N); **Taxon:** scientificName: Aphelocheirus freitagi; **Location:** island: Mindoro; country: Philippines; municipality: Roxas; locationRemarks: TR1f2; **Event:** eventDate: 21.08.2017

#### Distribution

**Figure 4.**
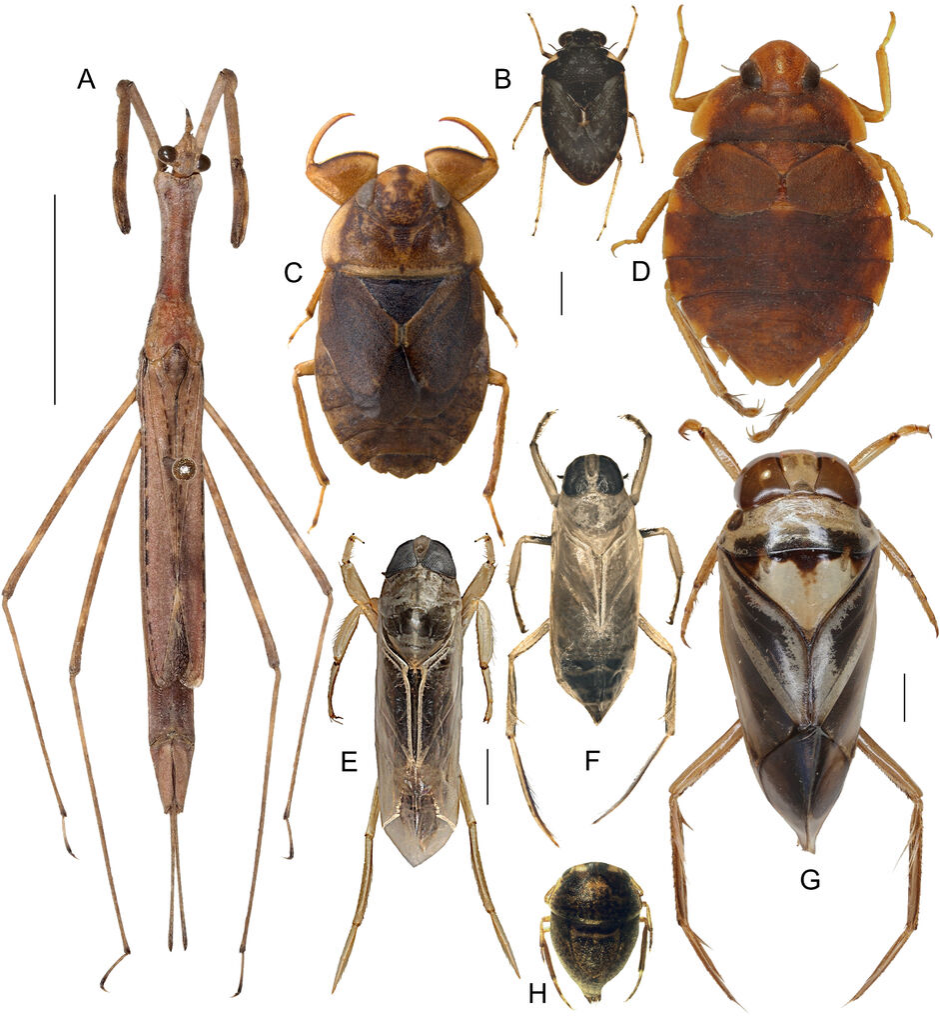
Habitus illustrations of Nepomorpha of Mindoro **A.** *Ranatra* sp. (*R. gracilis* group); **B.** *Ochterus polhemusi*; **C.** *Asthenocoris luzonensis paradisianus*; **D.** Aphelocheirus (s.str) freitagi; **E.** *Anisops kuroiwae*; **F.** *Anisops rhomboides*; **G.** *Enithares martini mindoroensis*; **H.** *Hydrotrephes stereoides mindoroensis*. Scale bars **A** 10 mm **B–H** 1 mm. **A** & **C** © NHMW Hemiptera Image Collection / photo: H. Bruckner, printed with permission.

**Figure 5.**
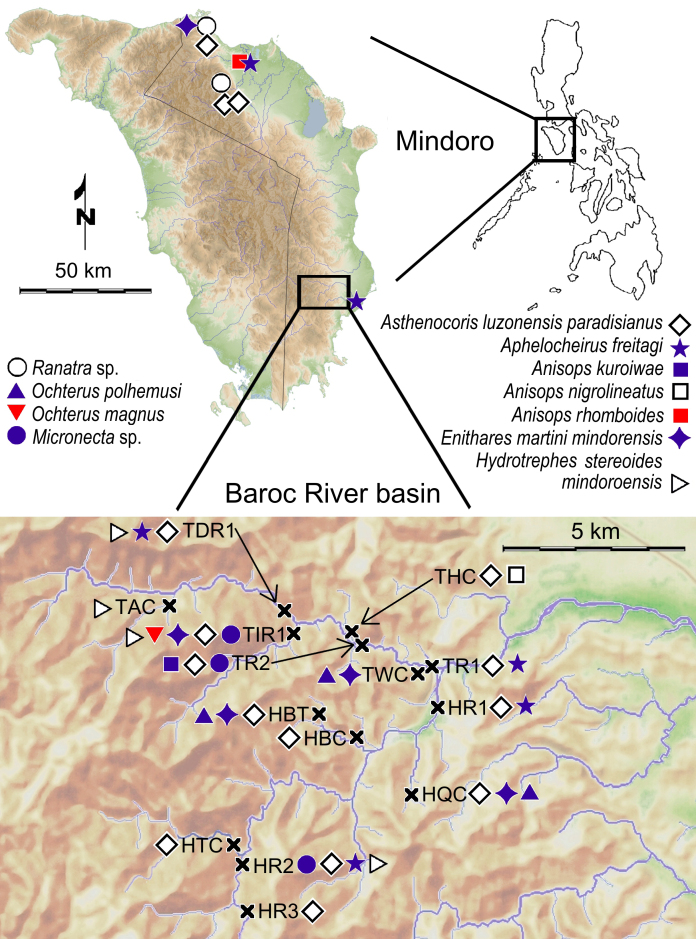
Distribution of the collecting sites of Nepomorpha material treated in this study.

*Aphelocheirus freitagi* Zettel and Pangantihon, 2010 (see Zettel and Pangantihon 2010) (Fig. 4D) is endemic to Mindoro and was previously only known from its type locality, Malayas River in Victoria, Oriental Mindoro (Zettel and Pangantihon 2010). Here we present the first records from Baco and Roxas (Fig. 5).

#### Taxon discussion

For identification, refer to Zettel and Pangantihon (2010).

#### Habitat

*Aphelocheirus* species Westwood, 1883 thrives in rather large, fast flowing rivers (Fig. 1D; Fig. 2A and B) with substrates consisting of a mixture of gravel and sand. The species is a typical benthic bottom dweller.

### Anisops kuroiwae

Matsumura, 1915

893A74AB-3951-52CC-8475-FAEC8983B375

#### Materials

- **Type status:** Other material. **Occurrence:** individualCount: 1 male (ma); **Taxon:** scientificName: Anisops kuroiwae; **Location:** island: Mindoro; country: Philippines; municipality: Roxas; locationRemarks: TR2(L); **Event:** eventDate: 03.07.2017

#### Distribution

The species (Fig. 4E) is known from Luzon, Catanduanes, Leyte, Mindoro (with an additional record, see Fig. 5), Negros, Palawan, Burias, Bohol, Panay, Samar, Siargao and Mindanao (Zettel et al. 2012). It is also a widespread species in the Oriental realm with records from India, southern China, Iriomote (off Japan), Melaka, and Batu Berendam, Malaysia (Nieser 2004).

#### Taxon discussion

For identification, refer to the key by Zettel (2003a) and the original description (Matsumura 1915).

#### Habitat

The single specimen was collected using a light trap not allowing for a specific microhabitat association. In general, representatives of this genus are found in isolated side pools (Fig. 3A) of streams and other stagnant water bodies (Zettel 2003a).

### Anisops nigrolineatus

Lundblad, 1933

95622298-D41B-57DE-AED1-785534B6F13B

#### Materials

- **Type status:** Other material. **Occurrence:** individualCount: 1 male (ma); **Taxon:** scientificName: Anisops nigrolineatus; **Location:** island: Mindoro; country: Philippines; municipality: Roxas; locationRemarks: THCt; **Event:** eventDate: 25.06.2017

#### Distribution

*Anisops nigrolineatus* Lundblad, 1933 (see Lundblad 1933) is widely distributed from India, Myanmar, Thailand, Brunei, Java and up to the Philippines (Zettel et al. 2012), with only a single previous record from Sibuyan Island (Nieser and Chen 1999). This is the first record from Mindoro Island (Fig. 5).

#### Taxon discussion

For identification, refer to the key by Zettel (2003a).

#### Habitat

The specimen was found in a small side pool near a slow-flowing stream.

### Anisops rhomboides

Nieser & Chen, 1999

14276503-3C24-5ED3-8AA6-04CD4CFC4E69

#### Materials

- **Type status:** Other material. **Occurrence:** individualCount: 4 males (ma), 3 (NN); **Taxon:** scientificName: Anisops rhomboides; **Location:** island: Mindoro; country: Philippines; municipality: Baco; locationRemarks: 353u1; **Event:** eventDate: 24.08.2017

#### Distribution

The species (Fig. 4F) is recorded from Brunei, Borneo, Sulawesi and the Philippines (Nieser and Chen 1999, Chen et al. 2005). Previous Philippine records refer to Leyte, Mindanao, Palawan and Tawi Tawi (Zettel et al. 2012). This is the first record from Mindoro (Fig. 5).

#### Taxon discussion

For identification, refer to the key by Zettel (2003a). Its specific epithet, *rhomboides*, refers to the lozenge-shaped fossa on the tylus (Nieser and Chen 1999), which is a good character for identification.

#### Habitat

*Anisops rhomboides* Nieser & Chen, 1999 (see Nieser and Chen 1999) is found in a variety of shallow, stagnant freshwater bodies, such as lakes, ponds, carabao puddles, marsh land and moats (Nieser and Chen 1999).

### Enithares martini mindoroensis

Nieser & Zettel, 1999

BD861D2C-85F6-5E28-9A3C-D97DBACB4E60

#### Materials

- **Type status:** Other material. **Occurrence:** individualCount: 1 female (ma); **Taxon:** scientificName: Enithares martini mindoroensis; **Location:** island: Mindoro; country: Philippines; municipality: Puerto Galera; locationRemarks: 304b; **Event:** eventDate: 07/25/2017
- **Type status:** Other material. **Occurrence:** individualCount: 1 female (ma); **Taxon:** scientificName: Enithares martini mindoroensis; **Location:** island: Mindoro; country: Philippines; municipality: Roxas; locationRemarks: HBTk; **Event:** eventDate: 09/21/2017
- **Type status:** Other material. **Occurrence:** individualCount: 6 males (ma); **Taxon:** scientificName: Enithares martini mindoroensis; **Location:** island: Mindoro; country: Philippines; municipality: Roxas; locationRemarks: HQCe; **Event:** eventDate: 06/30/2017
- **Type status:** Other material. **Occurrence:** individualCount: 4 males (ma); **Taxon:** scientificName: Enithares martini mindoroensis; **Location:** island: Mindoro; country: Philippines; municipality: Roxas; locationRemarks: HQCb; **Event:** eventDate: 06/30/2017
- **Type status:** Other material. **Occurrence:** individualCount: 1 female (ma); **Taxon:** scientificName: Enithares martini mindoroensis; **Location:** island: Mindoro; country: Philippines; municipality: Roxas; locationRemarks: TIRe; **Event:** eventDate: 07/10/2017
- **Type status:** Other material. **Occurrence:** individualCount: 1 female (ma); **Taxon:** scientificName: Enithares martini mindoroensis; **Location:** island: Mindoro; country: Philippines; municipality: Roxas; locationRemarks: TIRb; **Event:** eventDate: 09/22/2017
- **Type status:** Other material. **Occurrence:** individualCount: 2 males (ma); **Taxon:** scientificName: Enithares martini mindoroensis; **Location:** island: Mindoro; country: Philippines; municipality: Roxas; locationRemarks: TWCy; **Event:** eventDate: 06/25/2017
- **Type status:** Other material. **Occurrence:** individualCount: 1 female (ma); **Taxon:** scientificName: Enithares martini mindoroensis; **Location:** island: Mindoro; country: Philippines; municipality: Roxas; locationRemarks: TWCb; **Event:** eventDate: 09/23/2017

#### Distribution

This species (Fig. 4G) is widely distributed in the Philippines, but the subspecies is endemic to Mindoro (Nieser and Zettel 1999). See Fig. 5 for the new records.

#### Taxon discussion

For identification, refer to the key by Nieser and Zettel (1999).

#### Habitat

*Enithares martini mindoroensis* Nieser & Zettel, 1999 (see Nieser and Zettel 1999) can be found in calm shores and connected and isolated pools on the banks of streams (Nieser and Zettel 1999, current study).

### Hydrotrephes stereoides mindoroensis

Zettel, 2003

77CFE4BB-D1EE-5F48-8E6B-E577E26042CE

#### Materials

- **Type status:** Other material. **Occurrence:** individualCount: 13 males (ma); **Taxon:** scientificName: Hydrotrephes stereoides mindoroensis; **Location:** island: Mindoro; country: Philippines; municipality: Roxas; locationRemarks: HR2f; **Event:** eventDate: 07/03/2017
- **Type status:** Other material. **Occurrence:** individualCount: 2 males (ma); **Taxon:** scientificName: Hydrotrephes stereoides mindoroensis; **Location:** island: Mindoro; country: Philippines; municipality: Roxas; locationRemarks: TACb; **Event:** eventDate: 07/09/2017
- **Type status:** Other material. **Occurrence:** individualCount: 2 males (ma); **Taxon:** scientificName: Hydrotrephes stereoides mindoroensis; **Location:** island: Mindoro; country: Philippines; municipality: Roxas; locationRemarks: TDR1f; **Event:** eventDate: 09/22/2017
- **Type status:** Other material. **Occurrence:** individualCount: 3 males (ma); **Taxon:** scientificName: Hydrotrephes stereoides mindoroensis; **Location:** island: Mindoro; country: Philippines; municipality: Roxas; locationRemarks: TIR1b; **Event:** eventDate: 07/10/2017

#### Distribution

The subspecies (Fig. 4H) is endemic to Mindoro Island. See Fig. 5 for new records. Other *Hydrotrephes steroides* Zettel, 2003 (see Zettel 2003b) subspecies occur in north and central Luzon, namely ssp. montanus and ssp. steroides.

#### Taxon discussion

For identification, refer to the key by Zettel (2003b).

#### Habitat

*Hydrotrephes stereoides* Zettel, 2003 is mainly associated with lentic sections of running waters, swimming actively at the edges of plant material, rarely benthic in running waters. We found most specimens attached to wood in clean mountain rivers.

### Hebrus philippinus

Zettel, 2006

E160ED30-1B8D-56EE-A57F-E6191D0A0AC7

#### Materials

- **Type status:** Other material. **Occurrence:** individualCount: 1 female (ma); **Taxon:** scientificName: Hebrus philippinus; **Location:** island: Mindoro; country: Philippines; municipality: Puerto Galera; locationRemarks: 302j; **Event:** eventDate: 25.06.2017
- **Type status:** Other material. **Occurrence:** individualCount: 1 female (ma); **Taxon:** scientificName: Hebrus philippinus; **Location:** island: Mindoro; country: Philippines; municipality: Roxas; locationRemarks: HBTj; **Event:** eventDate: 05.07.2017
- **Type status:** Other material. **Occurrence:** individualCount: 5 males (ma); **Taxon:** scientificName: Hebrus philippinus; **Location:** island: Mindoro; country: Philippines; municipality: Roxas; locationRemarks: THFj; **Event:** eventDate: 07.07.2017
- **Type status:** Other material. **Occurrence:** individualCount: 7 males (ma); **Taxon:** scientificName: Hebrus philippinus; **Location:** island: Mindoro; country: Philippines; municipality: Roxas; locationRemarks: TIR1b; **Event:** eventDate: 22.09.2017
- **Type status:** Other material. **Occurrence:** individualCount: 1 female (ma); **Taxon:** scientificName: Hebrus philippinus; **Location:** island: Mindoro; country: Philippines; municipality: Roxas; locationRemarks: TR1j; **Event:** eventDate: 13.07.2017
- **Type status:** Other material. **Occurrence:** individualCount: 2 males (ma); **Taxon:** scientificName: Hebrus philippinus; **Location:** island: Mindoro; country: Philippines; municipality: Roxas; locationRemarks: TR2j; **Event:** eventDate: 07.07.2017
- **Type status:** Other material. **Occurrence:** individualCount: 3 males (ma); **Taxon:** scientificName: Hebrus philippinus; **Location:** island: Mindoro; country: Philippines; municipality: Roxas; locationRemarks: TR2j; **Event:** eventDate: 23.09.2017
- **Type status:** Other material. **Occurrence:** individualCount: 5 males (ma); **Taxon:** scientificName: Hebrus philippinus; **Location:** island: Mindoro; country: Philippines; municipality: Roxas; locationRemarks: TWCj; **Event:** eventDate: 23.09.2017

#### Distribution

The species is widely distributed in the Philippines, except for Palawan. This is the first record of this species in Mindoro (Fig. 7). It was previously recorded from Biliran, Bohol, Camiguin, Catanduanes, Cebu, Leyte, Luzon, Masbate, Mindanao, Negros, Panay, Polillo, Poro, Samar and Siquijor (Zettel 2006).

#### Taxon discussion

For identification, see Zettel (2006). The parameres have long setae both in the lateral and apical portions. Most of the species have straight parameres and only few were directed mesally. In contrast to the *Hebrus harrisi* complex, this species has a less distinct endocorium with only a small and elongate white spot. All specimens examined were macropterous.

#### Habitat

*Hebrus philippinus* Zettel, 2006 (see Zettel 2006) is quite euryoecious and can also thrive in anthropologically-disturbed habitats. It is commonly found on the banks of running waters and more rarely in nearby stagnant waters. Unlike other *Hebrus* species, it is often found in sunny and dry areas. We obtained most records from hygropetric microhabitats.

### Hebrus sp.

D039B35A-7129-5B7B-9885-49747E9E41E1

#### Materials

- **Type status:** Other material. **Occurrence:** individualCount: 1 female (ma); **Taxon:** scientificName: *Hebrus* sp; **Location:** island: Mindoro; country: Philippines; municipality: Roxas; locationRemarks: BR2b; **Event:** eventDate: 29.12.2017
- **Type status:** Other material. **Occurrence:** individualCount: 2 males (ma); **Taxon:** scientificName: *Hebrus* sp; **Location:** island: Mindoro; country: Philippines; municipality: Roxas; locationRemarks: HBCg; **Event:** eventDate: 30.06.2017
- **Type status:** Other material. **Occurrence:** individualCount: 1 female (ma); **Taxon:** scientificName: *Hebrus* sp; **Location:** island: Mindoro; country: Philippines; municipality: Roxas; locationRemarks: HBTb; **Event:** eventDate: 12.08.2017
- **Type status:** Other material. **Occurrence:** individualCount: 2 males (ma); **Taxon:** scientificName: *Hebrus* sp; **Location:** island: Mindoro; country: Philippines; municipality: Roxas; locationRemarks: HBT(E); **Event:** eventDate: 21.09.2017
- **Type status:** Other material. **Occurrence:** individualCount: 1 female (ma); **Taxon:** scientificName: *Hebrus* sp; **Location:** island: Mindoro; country: Philippines; municipality: Roxas; locationRemarks: HQCk; **Event:** eventDate: 21.09.2017
- **Type status:** Other material. **Occurrence:** individualCount: 5 males (ma); **Taxon:** scientificName: *Hebrus* sp; **Location:** island: Mindoro; country: Philippines; municipality: Roxas; locationRemarks: HQCb; **Event:** eventDate: 21.09.2017
- **Type status:** Other material. **Occurrence:** individualCount: 1 female (ma); **Taxon:** scientificName: *Hebrus* sp; **Location:** island: Mindoro; country: Philippines; municipality: Roxas; locationRemarks: HR1a; **Event:** eventDate: 01.07.2017
- **Type status:** Other material. **Occurrence:** individualCount: 1 female (ma); **Taxon:** scientificName: *Hebrus* sp; **Location:** island: Mindoro; country: Philippines; municipality: Roxas; locationRemarks: TIR1b; **Event:** eventDate: 22.09.2017

#### Taxon discussion

**Figure 6.**
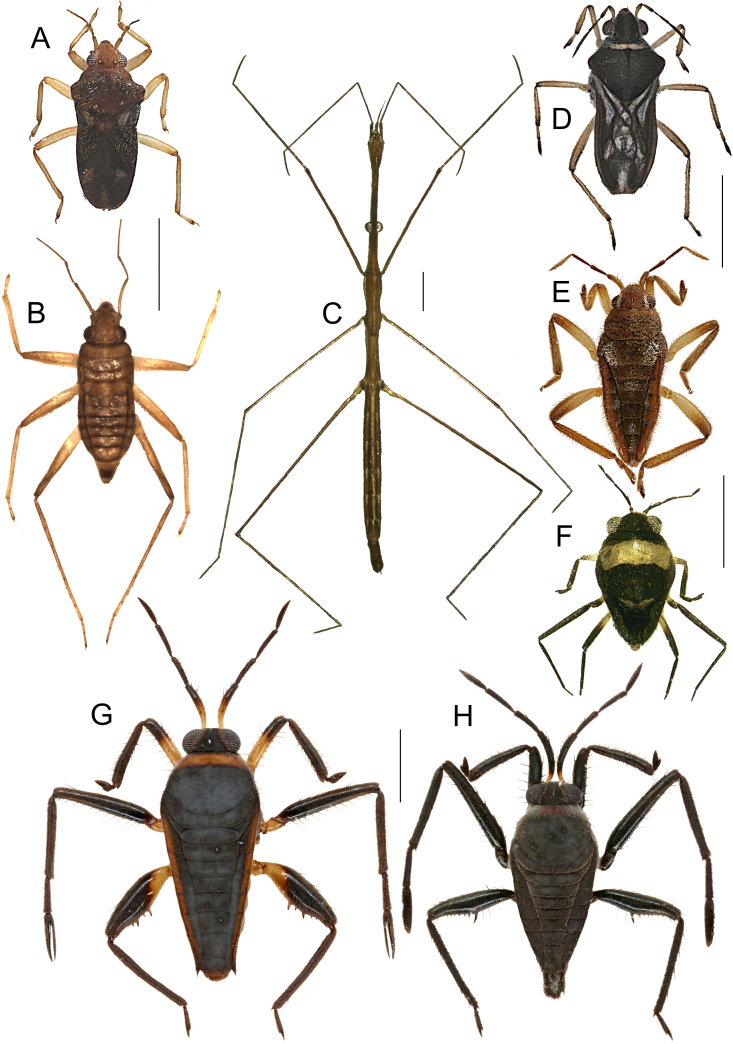
Selected habitus illustrations of Gerromorpha of Mindoro **A.** *Hebrus* sp.; **B.** *Mesovelia vittigera*; **C.** *Hydrometra mindoroensis*; **D.** *Microvelia douglasi*; **E.** Pseudovelia cf. curvata; **F.** *Strongylovelia mindoroensis*; **G.** *Rhagovelia mindoroensis*; **H.** *Rhagovelia raddai*. Scale bars 1 mm.

The *Hebrus harrisi* complex is discussed by Zettel (2004a), Zettel (2006). Due to the lack of specimens for comparison, our material (Fig. 6A) cannot be identified to species level. All specimens examined were macropterous.

#### Habitat

**Figure 7.**
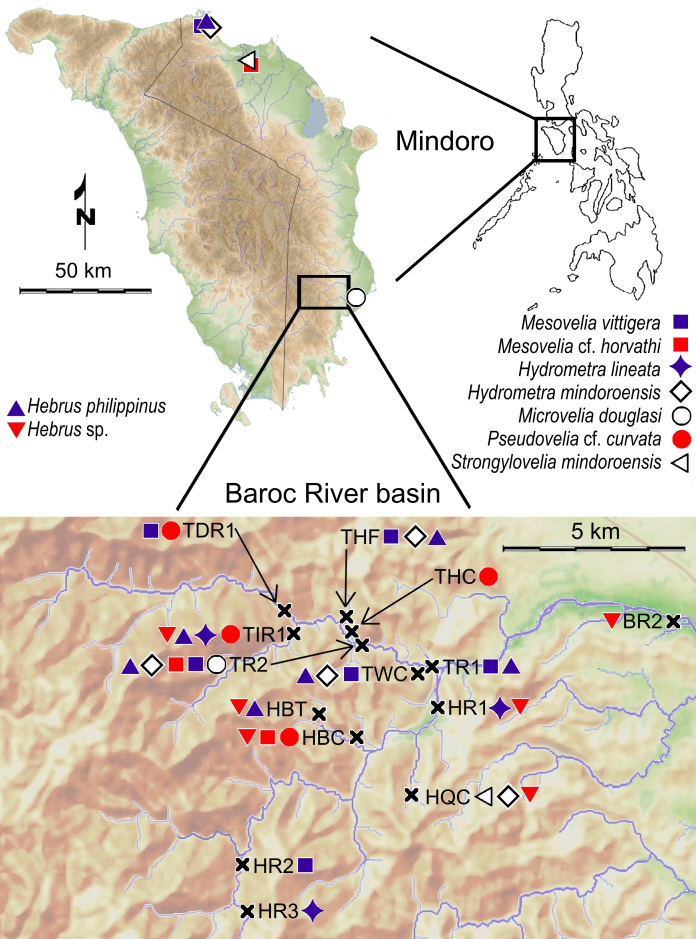
Distribution of the collecting sites of Gerromorpha material (part 1) treated in this study.

The specimens (Fig. 7) were found at the banks of more or less fast flowing sections of rivers of small to medium size.

### Mesovelia horvathi

Lundblad, 1933

E9060408-1B1D-5E31-9483-247930AC66C7

#### Materials

- **Type status:** Other material. **Occurrence:** individualCount: 1 female (ap); **Taxon:** scientificName: Mesovelia cf. horvathi; **Location:** island: Mindoro; country: Philippines; municipality: Baco; locationRemarks: 353u; **Event:** eventDate: 24.08.2017
- **Type status:** Other material. **Occurrence:** individualCount: 6 males (ap); **Taxon:** scientificName: Mesovelia cf. horvathi; **Location:** island: Mindoro; country: Philippines; municipality: Roxas; locationRemarks: HBCb; **Event:** eventDate: 08.07.2017
- **Type status:** Other material. **Occurrence:** individualCount: 1 female (ap); **Taxon:** scientificName: Mesovelia cf. horvathi; **Location:** island: Mindoro; country: Philippines; municipality: Roxas; locationRemarks: TR2j; **Event:** eventDate: 08.07.2017
- **Type status:** Other material. **Occurrence:** individualCount: 2 males (ap); **Taxon:** scientificName: Mesovelia cf. horvathi; **Location:** island: Mindoro; country: Philippines; municipality: Roxas; locationRemarks: TR2e; **Event:** eventDate: 10.08.2017
- **Type status:** Other material. **Occurrence:** individualCount: 1 female (ap); **Taxon:** scientificName: Mesovelia cf. horvathi; **Location:** island: Mindoro; country: Philippines; municipality: Roxas; locationRemarks: TR2b; **Event:** eventDate: 23.09.2017

#### Distribution

*Mesovelia horvathi* (s.l.) Lundblad, 1933 (see Lundblad 1933) was reported from Australia, China, India, Indonesia, Japan, Malaysia, Philippines (see Fig. 7 for new records), Sri Lanka, Singapore, Thailand, and Vietnam (Lundblad 1937, Chandra and Jehamalar 2011, Yang and Murphy 2011, Zettel 2014).

#### Taxon discussion

For identification, refer to the key by Yang and Murphy (2011). More recently, Jehamalar et al. (2019) have shown that *Mesovelia horvathi* consists of a complex of closely-related species. At least two species of this complex occur in the Philippines. Their correct names remain uncertain. As a result, we refrain from concluding that the previous records from other areas in the Philippines and specimens in this study are indeed *M. horvathi*.

#### Habitat

*Mesovelia horvathi* Lundblad, 1933 is common in plains and mountains in stagnant, slow flowing and even in brackish water (Yang and Murphy 2011). We found the species in similar, partly identical habitats like *Mesovelia vittigera*, but so far, never syntopic with the former species.

### Mesovelia vittigera

Horváth, 1895

75557FDA-8376-5DEC-9B8A-97335AAA3DDC

#### Materials

- **Type status:** Other material. **Occurrence:** individualCount: 1 female (ap); **Taxon:** scientificName: Mesovelia vittigera; **Location:** island: Mindoro; country: Philippines; municipality: Puerto Galera; locationRemarks: 305a; **Event:** eventDate: 24.06.2017
- **Type status:** Other material. **Occurrence:** individualCount: 1 female (ap); **Taxon:** scientificName: Mesovelia vittigera; **Location:** island: Mindoro; country: Philippines; municipality: Roxas; locationRemarks: HR2; **Event:** eventDate: 03.07.2017
- **Type status:** Other material. **Occurrence:** individualCount: 4 males (ap); **Taxon:** scientificName: Mesovelia vittigera; **Location:** island: Mindoro; country: Philippines; municipality: Roxas; locationRemarks: TDR1b; **Event:** eventDate: 05.07.2017
- **Type status:** Other material. **Occurrence:** individualCount: 4 males (ap); **Taxon:** scientificName: Mesovelia vittigera; **Location:** island: Mindoro; country: Philippines; municipality: Roxas; locationRemarks: THFj; **Event:** eventDate: 07.07.2017
- **Type status:** Other material. **Occurrence:** individualCount: 11 female (ap); **Taxon:** scientificName: Mesovelia vittigera; **Location:** island: Mindoro; country: Philippines; municipality: Roxas; locationRemarks: TR1b; **Event:** eventDate: 13.07.2017
- **Type status:** Other material. **Occurrence:** individualCount: 1 female (ap); **Taxon:** scientificName: Mesovelia vittigera; **Location:** island: Mindoro; country: Philippines; municipality: Roxas; locationRemarks: TR2j; **Event:** eventDate: 23.09.2017
- **Type status:** Other material. **Occurrence:** individualCount: 2 males (ap); **Taxon:** scientificName: Mesovelia vittigera; **Location:** island: Mindoro; country: Philippines; municipality: Roxas; locationRemarks: TWCb; **Event:** eventDate: 06.07.2017
- **Type status:** Other material. **Occurrence:** individualCount: 2 males (ap); **Taxon:** scientificName: Mesovelia vittigera; **Location:** island: Mindoro; country: Philippines; municipality: Roxas; locationRemarks: TWCj; **Event:** eventDate: 23.09.2017

#### Distribution

The species (Fig. 6B) is widely distributed in the tropics and subtropics of the Old World, including the Philippines (Zettel 2014). See Fig. 7 for additional records.

#### Taxon discussion

For identification, refer to the key by Yang and Murphy (2011). All specimens examined were apterous.

#### Habitat

*Mesovelia* species are commonly found amongst marginal vegetation in standing waters of ponds and streams. *Mesovelia vittigera* Horváth, 1895 (see Horváth 1895) can also be found in brackish-water habitats (Yang and Murphy 2011). We found several specimens on shaded, wet rocks and at the stream littoral with mineral substrates (Fig. 1B).

### Hydrometra lineata

Eschscholtz, 1822

389B5EBF-D7C2-5F3F-8E8A-A733ABA4D1E6

#### Materials

- **Type status:** Other material. **Occurrence:** individualCount: 1 female (ma); **Taxon:** scientificName: Hydrometra lineata; **Location:** island: Mindoro; country: Philippines; municipality: Roxas; locationRemarks: HR1b; **Event:** eventDate: 01.07.2017
- **Type status:** Other material. **Occurrence:** individualCount: 1 female (ma); **Taxon:** scientificName: Hydrometra lineata; **Location:** island: Mindoro; country: Philippines; municipality: Roxas; locationRemarks: HR3y; **Event:** eventDate: 21.06.2017
- **Type status:** Other material. **Occurrence:** individualCount: 2 males (ma); **Taxon:** scientificName: Hydrometra lineata; **Location:** island: Mindoro; country: Philippines; municipality: Roxas; locationRemarks: TIR1b; **Event:** eventDate: 30.06.2017

#### Distribution

*Hydrometra lineata* Eschscholtz, 1822 (see Eschscholtz 1822) is widely distributed in the Philippines (see Fig. 7 for new records), the Oriental Realm, Wallacea and New Guinea (Polhemus and Lansbury 1997, Gapud et al. 2003).

#### Taxon discussion

For identification, refer to the key by Gapud et al. (2003). All specimens examined were macropterous.

#### Habitat

The species is often found in large stagnant water bodies and rarely seen in running waters; however, all our samples are from stream banks.

### Hydrometra mindoroensis

Polhemus, 1976

319A7012-BF23-5945-8981-C44B68403179

#### Materials

- **Type status:** Other material. **Occurrence:** individualCount: 1 female (ma); **Taxon:** scientificName: Hydrometra mindoroensis; **Location:** island: Mindoro; country: Philippines; municipality: Puerto Galera; locationRemarks: 305h; **Event:** eventDate: 24.06.2017
- **Type status:** Other material. **Occurrence:** individualCount: 1 female (ap); **Taxon:** scientificName: Hydrometra mindoroensis; **Location:** island: Mindoro; country: Philippines; municipality: Roxas; locationRemarks: HQCy; **Event:** eventDate: 30.06.2017
- **Type status:** Other material. **Occurrence:** individualCount: 5 males (ap, ma); **Taxon:** scientificName: Hydrometra mindoroensis; **Location:** island: Mindoro; country: Philippines; municipality: Roxas; locationRemarks: HQCb; **Event:** eventDate: 21.09.2017
- **Type status:** Other material. **Occurrence:** individualCount: 1 female (ap); **Taxon:** scientificName: Hydrometra mindoroensis; **Location:** island: Mindoro; country: Philippines; municipality: Roxas; locationRemarks: THFj; **Event:** eventDate: 07.07.2017
- **Type status:** Other material. **Occurrence:** individualCount: 1 female (ma); **Taxon:** scientificName: Hydrometra mindoroensis; **Location:** island: Mindoro; country: Philippines; municipality: Roxas; locationRemarks: TR2j; **Event:** eventDate: 08.07.2017
- **Type status:** Other material. **Occurrence:** individualCount: 1 female (ap); **Taxon:** scientificName: Hydrometra mindoroensis; **Location:** island: Mindoro; country: Philippines; municipality: Roxas; locationRemarks: TWC2y; **Event:** eventDate: 25.06.2017

#### Distribution

*Hydrometra mindoroensis* Polhemus, 1976 (in Polhemus and Reisen 1976) (Fig. 6C) is a widespread species in the Philippines and neighbouring areas (Borneo, Sulawesi, New Guinea) (Polhemus and Lansbury 1997, Gapud et al. 2003).

#### Taxon discussion

For identification, refer to the key by Gapud et al. (2003).

#### Habitat

This species can be found in both stagnant waters and edges of streams and rivers (Gapud et al. 2003). We found the species in shaded hygropetric sites and at stream banks with mineral and organic substrates (Fig. 1B).

### Microvelia douglasi

Scott, 1874

E3D61488-A5E2-57FF-B7AB-FCA2AB95FC7D

#### Materials

- **Type status:** Other material. **Occurrence:** individualCount: 1 female (ap); **Taxon:** scientificName: Microvelia douglasi; **Location:** island: Mindoro; country: Philippines; municipality: Roxas; locationRemarks: BR3m; **Event:** eventDate: 08.07.2017
- **Type status:** Other material. **Occurrence:** individualCount: 10 males (ma), 18 males (ap); **Taxon:** scientificName: Microvelia douglasi; **Location:** island: Mindoro; country: Philippines; municipality: Roxas; locationRemarks: TR2e; **Event:** eventDate: 23.09.2017

#### Distribution

This species (Fig. 6D), which was originally described from Japan (Scott 1874), is widely distributed in the Oriental, Australian and Melanesian Regions reaching eastwards to the remote islands of the West Pacific Region (Andersen and Weir 2003). See Fig. 7 for the additional records.

#### Taxon discussion

See Andersen et al. (2002) for identification. The species is rarely collected in the Island.

#### Habitat

The species was found on stream banks, specifically in side pools (Fig. 3B), such as at site BR3 (Fig. 2A).

### Pseudovelia curvata

Hecher, 2006

7D1A3A31-6FCA-5453-9F33-F8E18D3E1779

#### Materials

- **Type status:** Other material. **Occurrence:** individualCount: 2 males (ap); **Taxon:** scientificName: Pseudovelia cf. curvata; **Location:** island: Mindoro; country: Philippines; municipality: Roxas; locationRemarks: HBCg; **Event:** eventDate: 30.06.2017
- **Type status:** Other material. **Occurrence:** individualCount: 1 female (ap); **Taxon:** scientificName: Pseudovelia cf. curvata; **Location:** island: Mindoro; country: Philippines; municipality: Roxas; locationRemarks: TDR1h; **Event:** eventDate: 04.07.2017
- **Type status:** Other material. **Occurrence:** individualCount: 1 female (ap); **Taxon:** scientificName: Pseudovelia cf. curvata; **Location:** island: Mindoro; country: Philippines; municipality: Roxas; locationRemarks: THCe; **Event:** eventDate: 07.07.2017
- **Type status:** Other material. **Occurrence:** individualCount: 6 males (ap); **Taxon:** scientificName: Pseudovelia cf. curvata; **Location:** island: Mindoro; country: Philippines; municipality: Roxas; locationRemarks: TIR1b; **Event:** eventDate: 22.09.2017

#### Distribution

The typical form is only known from the Mountain Province in northern Luzon (Hecher 2006). See Fig. 7 for the collecting sites of the slightly varying Mindoro material.

#### Taxon discussion

The specimens (Fig. 6E) closely resemble those of *Pseudovelia curvata* Hecher, 2006 following the key in Hecher (2006), displaying the following characters: first metatarsal segment devoid of a tuft of very long setae basally; first and second metatarsal segment with a row of long setae over entire length; and metatarsus about half as long as metatibia. However, the pygophore of males has long, bristle-like setae on its caudo-lateral margin like *P. gapudi* Hecher, 2006. Given the lack of any records, except for the specimens in hand and the type material from northern Luzon, it must remain unsolved if the material is conspecific or represents a new, but related species. This variation is only recognised in our specimens from Mindoro.

#### Habitat

The specimens were found at banks of creeks and rivers, both in calm and flowing sections.

### Strongylovelia mindoroensis

Lansbury & Zettel, 1997

3A122150-3696-5528-8B86-E2CC8EBD1111

#### Materials

- **Type status:** Other material. **Occurrence:** individualCount: 3 males (ma); **Taxon:** scientificName: Strongylovelia mindoroensis; **Location:** island: Mindoro; country: Philippines; municipality: Baco; locationRemarks: 353u; **Event:** eventDate: 24.08.2017
- **Type status:** Other material. **Occurrence:** individualCount: 2 males (ma, ap); **Taxon:** scientificName: Strongylovelia mindoroensis; **Location:** island: Mindoro; country: Philippines; municipality: Roxas; locationRemarks: HQCy; **Event:** eventDate: 21.09.2017

#### Distribution

*Strongylovelia mindoroensis* Lansbury & Zettel, 1997 (see Lansbury and Zettel 1997) (Fig. 6F) is endemic to Mindoro and only known from the type locality in Puerto Galera (Lansbury and Zettel 1997) and our records from Baco and Roxas (Fig. 7).

#### Taxon discussion

For identification, refer to the key by Zettel (2003c).

#### Habitat

The species was found in slow flowing water and a residual pool with floating plants.

### Rhagovelia mindoroensis

Zettel, 1994

D3008ACE-CC9E-563C-A12E-37299BF26F81

#### Materials

- **Type status:** Other material. **Occurrence:** individualCount: 2 males (ap); **Taxon:** scientificName: Rhagovelia mindoroensis; **Location:** island: Mindoro; country: Philippines; municipality: Puerto Galera; locationRemarks: 304b; **Event:** eventDate: 24.06.2017
- **Type status:** Other material. **Occurrence:** individualCount: 1 female (ma); **Taxon:** scientificName: Rhagovelia mindoroensis; **Location:** island: Mindoro; country: Philippines; municipality: Roxas; locationRemarks: HR3e; **Event:** eventDate: 19.06.2017
- **Type status:** Other material. **Occurrence:** individualCount: 2 males; **Taxon:** scientificName: Rhagovelia mindoroensis; **Location:** island: Mindoro; country: Philippines; municipality: Roxas; locationRemarks: TDR1y; **Event:** eventDate: 23.06.2017
- **Type status:** Other material. **Occurrence:** individualCount: 1 female (ap); **Taxon:** scientificName: Rhagovelia mindoroensis; **Location:** island: Mindoro; country: Philippines; municipality: Roxas; locationRemarks: TDR4y; **Event:** eventDate: 04.07.2017
- **Type status:** Other material. **Occurrence:** individualCount: 1 female (ma); **Taxon:** scientificName: Rhagovelia mindoroensis; **Location:** island: Mindoro; country: Philippines; municipality: Roxas; locationRemarks: THCy; **Event:** eventDate: 29.06.2017
- **Type status:** Other material. **Occurrence:** individualCount: 1 female (ma); **Taxon:** scientificName: Rhagovelia mindoroensis; **Location:** island: Mindoro; country: Philippines; municipality: Roxas; locationRemarks: TWCt; **Event:** eventDate: 25.06.2017

#### Distribution

**Figure 8.**
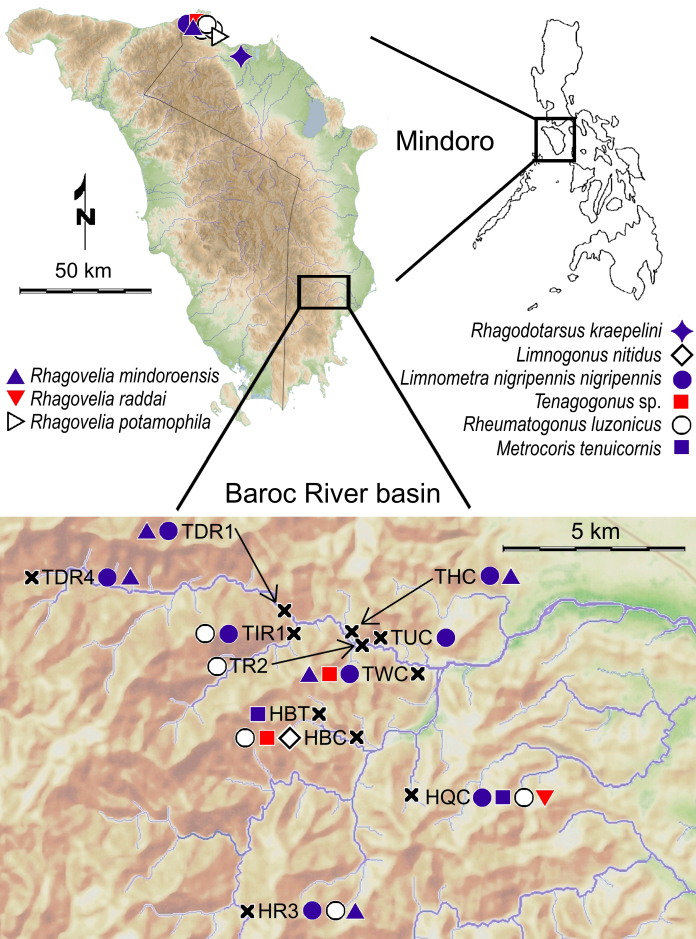
Distribution of the collecting sites of Gerromorpha material (part 2) treated in this study.

*Rhagovelia mindoroensis* (Fig. 6G) is an endemic species in Mindoro (Zettel 1994). See Fig. 8 for our additional records.

#### Taxon discussion

For identification, see Zettel (1994).

#### Habitat

*Rhagovelia mindoroensis* Zettel, 1994 (see Zettel 1994) is usually found in secondary forests and in anthropogenic terrains close to the coast. They particularly inhabit still waters sections of streams (Zettel 1994). We also found it in small side pools of medium-sized rivers and creeks. The collection at site TDR4 (Fig. 2C) at 700 m altitude is surprising.

### Rhagovelia raddai

Zettel, 1994

48C781DC-C9C4-59A2-8620-DDB10DA8140A

#### Materials

- **Type status:** Other material. **Occurrence:** individualCount: 4 males (ap); **Taxon:** scientificName: Rhagovelia raddai; **Location:** island: Mindoro; country: Philippines; municipality: Roxas; locationRemarks: HQCz; **Event:** eventDate: 30.06.2017
- **Type status:** Other material. **Occurrence:** individualCount: 4 males (ma); **Taxon:** scientificName: Rhagovelia raddai; **Location:** island: Mindoro; country: Philippines; municipality: Roxas; locationRemarks: HQCy; **Event:** eventDate: 23.09.2017

#### Distribution

*Rhagovelia raddai* Zettel, 1994 (see Zettel 1994) (Fig. 6H) is endemic to Mindoro (Zettel 1994). See Fig. 8 for additional records.

#### Taxon discussion

For identification, see Zettel (1994).

#### Habitat

The species is commonly found in moderately fast flowing creeks and lotic sections of the river (Zettel 1994), such as in our study.

### Rhagovelia potamophila

Zettel, 1996

5E8DA30D-6C18-5472-AC3D-75C018D0184A

#### Materials

- **Type status:** Other material. **Occurrence:** individualCount: 2 males (ma); **Taxon:** scientificName: Rhagovelia potamophila; **Location:** island: Mindoro; country: Philippines; municipality: Puerto Galera; locationRemarks: 305z; **Event:** eventDate: 22.06.2017

#### Distribution

*Rhagovelia potamophila* Zettel, 1996 (see Zettel 1996) is endemic to Mindoro (Freitag and Pangantihon 2010). We present one additional record (Fig. 8).

#### Taxon discussion

For identification, see Zettel (1996), a habitus illustration is provided in Freitag and Pangantihon (2010).

#### Habitat

The specimens were found neustic on flowing water near root packs of a small river in a rural area (Fig. 1B).

### Rhagadotarsus (Rhagadotarsus) kraepelini

Breddin, 1905

A37DEA25-9529-5B37-BEC6-7C9D85EBD799

#### Materials

- **Type status:** Other material. **Occurrence:** individualCount: 4 males (ap); **Taxon:** scientificName: Rhagadotarsus (Rhagadotarsus) kraepelini; **Location:** island: Mindoro; country: Philippines; municipality: Baco; locationRemarks: 353u1; **Event:** eventDate: 24.08.2017

#### Distribution

Rhagadotarsus (Rhagadotarsus) kraepelini Breddin, 1905 (see Breddin 1905) is a widespread species in southern and south-eastern Asia and Micronesia (Polhemus and Karunaratne 1993). In the Philippines, *R. kraepelini* is distributed throughout the country, but the limits of its distribution in the Philippines are still unclear (Zettel 2014). See Fig. 8 for the additional record.

#### Taxon discussion

For identification, refer to the key by Polhemus and Karunaratne (1993).

#### Habitat

The specimens were found amongst floating water plants in a residual pool of a dried-up lowland creek in a rural area.

### Limnogonus nitidus

(Mayr, 1865)

9341040E-4DB8-58EB-A131-529E30732405

#### Materials

- **Type status:** Other material. **Occurrence:** individualCount: 3 males (ap), 4 males (ma); **Taxon:** scientificName: Limnogonus nitidus; **Location:** island: Mindoro; country: Philippines; municipality: Roxas; locationRemarks: HBCy; **Event:** eventDate: 08.07.2017

#### Distribution

**Figure 9.**
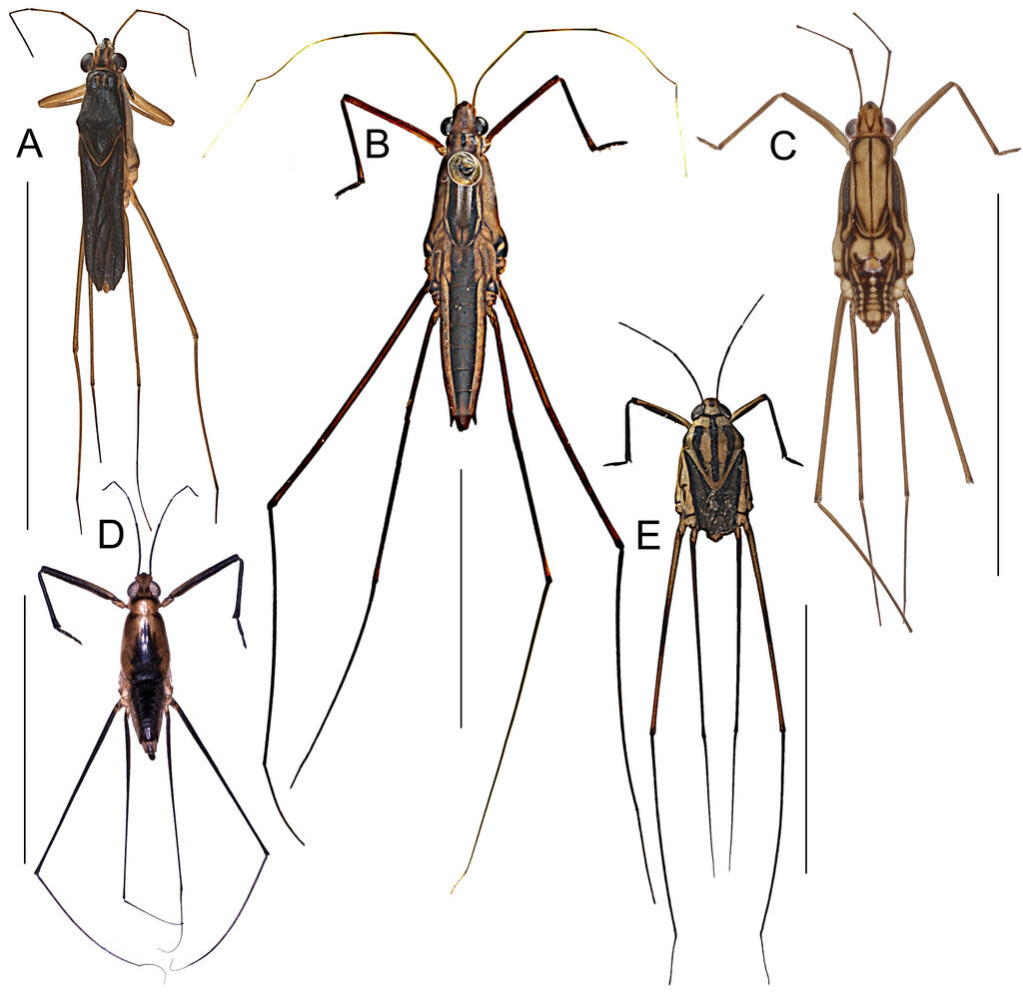
Selected habitus illustrations of Gerromorpha of Mindoro **A.** *Limnogonus nitidus*; **B.** *Limnometra nigripennis nigripennis*; **C.** *Tenagogonus* sp. (mutilated specimen); **D.** *Rheumatogonus luzonicus*; **E.** *Metrocoris tenuicornis*. Scale bars 10 mm.

The species (Fig. 9A) is widespread in the Philippines, Maldives, India, Sri Lanka, southern China and Indonesia (Damgaard et al. 2014), with the first records from Cambodia recently documented by Zettel et al. (2017). See Fig. 8 for our additional records.

#### Taxon discussion

Refer to Cheng et al. (2001) for the identification.

#### Habitat

In the Oriental realm, most species of *Limnogonus* Stål, 1868 prefer sheltered places in standing waters, which makes them somewhat gregarious. *Limnogonus nitidus* (Mayr, 1865) (see Mayr 1865) and *L. fossarum* (Fabricius, 1775) (see Fabricius 1775) are probably the only Oriental species of the genus that successfully colonise intermittent habitats.

### Limnometra nigripennis nigripennis

Mayr, 1865

E9CE3AAE-5019-50EE-BDC4-AE864C7A3DEE

#### Materials

- **Type status:** Other material. **Occurrence:** individualCount: 1 female (ap); **Taxon:** scientificName: Limnometra nigripennis nigripennis; **Location:** island: Mindoro; country: Philippines; municipality: Puerto Galera; locationRemarks: 304b; **Event:** eventDate: 07/24/2017
- **Type status:** Other material. **Occurrence:** individualCount: 2 males (ma); **Taxon:** scientificName: Limnometra nigripennis nigripennis; **Location:** island: Mindoro; country: Philippines; municipality: Roxas; locationRemarks: HQCb; **Event:** eventDate: 06/30/2017
- **Type status:** Other material. **Occurrence:** individualCount: 3 males (ma), 1 female (ap); **Taxon:** scientificName: Limnometra nigripennis nigripennis; **Location:** island: Mindoro; country: Philippines; municipality: Roxas; locationRemarks: HQCc; **Event:** eventDate: 06/30/2017
- **Type status:** Other material. **Occurrence:** individualCount: 3 males (ap); **Taxon:** scientificName: Limnometra nigripennis nigripennis; **Location:** island: Mindoro; country: Philippines; municipality: Roxas; locationRemarks: TDR1b; **Event:** eventDate: 06/23/2017
- **Type status:** Other material. **Occurrence:** individualCount: 1 female (ap); **Taxon:** scientificName: Limnometra nigripennis nigripennis; **Location:** island: Mindoro; country: Philippines; municipality: Roxas; locationRemarks: TDR4c; **Event:** eventDate: 07/04/2017
- **Type status:** Other material. **Occurrence:** individualCount: 1 female (ap); **Taxon:** scientificName: Limnometra nigripennis nigripennis; **Location:** island: Mindoro; country: Philippines; municipality: Roxas; locationRemarks: THCb; **Event:** eventDate: 06/25/2017
- **Type status:** Other material. **Occurrence:** individualCount: 1 female (ma); **Taxon:** scientificName: Limnometra nigripennis nigripennis; **Location:** island: Mindoro; country: Philippines; municipality: Roxas; locationRemarks: THCb; **Event:** eventDate: 06/29/2017
- **Type status:** Other material. **Occurrence:** individualCount: 5 males (ap), 1 female (ap); **Taxon:** scientificName: Limnometra nigripennis nigripennis; **Location:** island: Mindoro; country: Philippines; municipality: Roxas; locationRemarks: TIR1b; **Event:** eventDate: 09/22/2017
- **Type status:** Other material. **Occurrence:** individualCount: 4 males (ma); **Taxon:** scientificName: Limnometra nigripennis nigripennis; **Location:** island: Mindoro; country: Philippines; municipality: Roxas; locationRemarks: HR3e; **Event:** eventDate: 06/19/2017
- **Type status:** Other material. **Occurrence:** individualCount: 1 female (ap); **Taxon:** scientificName: Limnometra nigripennis nigripennis; **Location:** island: Mindoro; country: Philippines; municipality: Roxas; locationRemarks: TUCb; **Event:** eventDate: 11/17/2017
- **Type status:** Other material. **Occurrence:** individualCount: 2 males (ma); **Taxon:** scientificName: Limnometra nigripennis nigripennis; **Location:** island: Mindoro; country: Philippines; municipality: Roxas; locationRemarks: TWCt; **Event:** eventDate: 06/25/2017
- **Type status:** Other material. **Occurrence:** individualCount: 3 males (ap); **Taxon:** scientificName: Limnometra nigripennis nigripennis; **Location:** island: Mindoro; country: Philippines; municipality: Roxas; locationRemarks: TWCb; **Event:** eventDate: 09/23/2017

#### Distribution

*Limnometra nigripennis nigripennis* Mayr, 1865 (see *Mayr 1865*) (Fig. 9B) is endemic and widespread in the Philippines. Records are available from Biliran, Bohol, Camiguin, Guimaras, Leyte, Luzon, Mindanao, Mindoro (see Fig. 8 for our additional records), Negros, Panay, Polillo, Sibuyan, Tablas, Ticao, as well as unpublished records from Masbate (Zettel 2014 and references therein).

#### Taxon discussion

For identification, refer to Zettel (2004b).

#### Habitat

*Limnometra nigripennis* Mayr 1865 is amongst the most widespread and abundant Gerromorpha of Philippine running waters (Zettel 2004b, Zettel 2014). It exhibits higher tolerance to environmental disturbances in streams than other species. However, we recorded it predominantely from clean streams (Fig. 2C, D). Lentic sections of very small to medium-sized streams and pools associated with running water are its typical habitats (Zettel 2014).

### Tenagogonus sp.

33A024D9-48D1-54ED-8C94-13777D73E6DE

#### Materials

- **Type status:** Other material. **Occurrence:** individualCount: 2 males (ap); **Taxon:** scientificName: *Tenagogonus* sp.; **Location:** island: Mindoro; country: Philippines; municipality: Puerto Galera; locationRemarks: 301y; **Event:** eventDate: 22.06.2017
- **Type status:** Other material. **Occurrence:** individualCount: 1 female (ap); **Taxon:** scientificName: *Tenagogonus* sp.; **Location:** island: Mindoro; country: Philippines; municipality: Roxas; locationRemarks: HBCy; **Event:** eventDate: 08.07.2017
- **Type status:** Other material. **Occurrence:** individualCount: 1 female (ap); **Taxon:** scientificName: *Tenagogonus* sp.; **Location:** island: Mindoro; country: Philippines; municipality: Roxas; locationRemarks: TWCy; **Event:** eventDate: 25.06.2017

#### Taxon discussion

This genus is in need of revision (Zettel 2014). The specimens (Fig. 9C) probably belong to a new species related to *Tenagogonus bergrothi* Hungerford & Matsuda, 1958 (see Hungerford and Matsuda 1958), from Luzon. The genus is widespread throughout the Afrotropical, Oriental and Australian Regions, extending eastwards up to Fiji (Damgaard et al. 2014). Fifteen species are known in the Malesian Region, including the Philippines (Chen et al. 2005).

#### Habitat

The collected specimens were found in small forest streams, a common habitat of representatives of the genus.

### Rheumatogonus luzonicus

(Kirkaldy, 1909)

B8310F72-DA2B-54E7-A7F2-D3F3EBA69ACE

#### Materials

- **Type status:** Other material. **Occurrence:** individualCount: 4 males (ap); **Taxon:** scientificName: Rheumatogonus luzonicus; **Location:** island: Mindoro; country: Philippines; municipality: Puerto Galera; locationRemarks: 301y; **Event:** eventDate: 22.06.2017
- **Type status:** Other material. **Occurrence:** individualCount: 4 males (ap), 1 female (ap); **Taxon:** scientificName: Rheumatogonus luzonicus; **Location:** island: Mindoro; country: Philippines; municipality: Puerto Galera; locationRemarks: 305y; **Event:** eventDate: 24.06.2017
- **Type status:** Other material. **Occurrence:** individualCount: 2 males (ap); **Taxon:** scientificName: Rheumatogonus luzonicus; **Location:** island: Mindoro; country: Philippines; municipality: Puerto Galera; locationRemarks: 396y; **Event:** eventDate: 02.07.2017
- **Type status:** Other material. **Occurrence:** individualCount: 1 female (ap); **Taxon:** scientificName: Rheumatogonus luzonicus; **Location:** island: Mindoro; country: Philippines; municipality: Roxas; locationRemarks: HBCb; **Event:** eventDate: 08.07.2017
- **Type status:** Other material. **Occurrence:** individualCount: 6 males (ap); **Taxon:** scientificName: Rheumatogonus luzonicus; **Location:** island: Mindoro; country: Philippines; municipality: Roxas; locationRemarks: HQCz; **Event:** eventDate: 30.06.2017
- **Type status:** Other material. **Occurrence:** individualCount: 3 males (ap); **Taxon:** scientificName: Rheumatogonus luzonicus; **Location:** island: Mindoro; country: Philippines; municipality: Roxas; locationRemarks: HQCy; **Event:** eventDate: 30.06.2017
- **Type status:** Other material. **Occurrence:** individualCount: 7 males (ap); **Taxon:** scientificName: Rheumatogonus luzonicus; **Location:** island: Mindoro; country: Philippines; municipality: Roxas; locationRemarks: HR3b; **Event:** eventDate: 31.06.2017
- **Type status:** Other material. **Occurrence:** individualCount: 1 female (ap); **Taxon:** scientificName: Rheumatogonus luzonicus; **Location:** island: Mindoro; country: Philippines; municipality: Roxas; locationRemarks: TIRb; **Event:** eventDate: 22.09.2017
- **Type status:** Other material. **Occurrence:** individualCount: 6 males (ap); **Taxon:** scientificName: Rheumatogonus luzonicus; **Location:** island: Mindoro; country: Philippines; municipality: Roxas; locationRemarks: TR2y; **Event:** eventDate: 25.02.2017

#### Distribution

*Rheumatogonus luzonicus* (Kirkaldy, 1909) (see Kirkaldy 1909) (Fig. 9D) is endemic to the Philippines and has been recorded from Luzon, Mindanao, Mindoro (Freitag and Pangantihon 2010, see Fig. 8 for additional records), Negros and Panay (Chen and Nieser 2002). Some unpublished records from Catanduanes, Marinduque, Sibuyan, Ticao, Cebu, Samar, Leyte, Mindanao, Siquijor and Poro have also been recognised belonging to this species (Zettel 2014).

#### Taxon discussion

For identification, refer to Chen and Nieser (2002).

#### Habitat

The specimens were collected from the surface and littoral of calm and moderately fast flowing creeks and medium-sized rivers (Fig. 1A, B). In general, *Rheumatogonus* Kirkaldy, 1909 species inhabit mountain streams, creeks and waterfalls in the Oriental Region, specifically the shaded, steady slow-lotic sections of such watercourses (Chen et al. 2005).

### Metrocoris tenuicornis

Esaki, 1926

AA805018-22B7-5516-A832-EA33793531EE

#### Materials

- **Type status:** Other material. **Occurrence:** individualCount: 1 female (ap); **Taxon:** scientificName: Metrocoris tenuicornis; **Location:** island: Mindoro; country: Philippines; municipality: Roxas; locationRemarks: HBTb; **Event:** eventDate: 12.08.2017
- **Type status:** Other material. **Occurrence:** individualCount: 3 males (ap, ma); **Taxon:** scientificName: Metrocoris tenuicornis; **Location:** island: Mindoro; country: Philippines; municipality: Roxas; locationRemarks: HQCy; **Event:** eventDate: 21.09.2017

#### Distribution

*Metrocoris tenuicornis* Esaki, 1926 (see Esaki 1926) (Fig. 9E) is a widspread species in western India, southern China, mainland southeast Asia, Sumatra, Java and Borneo, but only known so far from Mindoro and Greater Palawan, in the Philippines (Chen and Nieser 1993, Freitag and Zettel 2012).

#### Taxon discussion

For identification, refer to Chen and Nieser (1993).

#### Habitat

Quiet bays of smoothly-flowing streams and the edge of large rocks in the middle of streams are the preferred habitats of *Metrocoris* Mayr, 1865 species (Chen and Nieser 1993), such as the records presented here (Fig. 8).

## Checklists

### Checklist of the Nepomorpha of Mindoro

#### Ranatra

Fabricius, 1790

9679C066-190B-5142-B0D7-1AEE8F053340

##### Distribution

Philippine-endemic

##### Notes

undescribed species

#### Ochterus polhemusi

Gapud, 1981

1E19F2BC-96DA-5A77-B96F-DE285F0EF8F9

https://www.gbif.org/species/9451187

##### Distribution

Philippine-endemic

#### Ochterus magnus

Gapud & San Valentin, 1977

6E2710AC-28B0-5BF4-B570-9EC5371B112F

https://www.gbif.org/species/9564741

##### Distribution

Philippine-endemic

##### Notes

new Mindoro record

#### Ochterus marginatus insularis

Rieger, 1977

49088337-B1BA-52F7-8BF7-FB4D8FD5BD87

https://www.gbif.org/species/9636304

##### Distribution

Philippine-endemic

#### Ochterus philippinensis

Gapud, 1977

6A82C8A2-AB56-5403-BDF4-ED094200C969

https://www.gbif.org/species/9344209

##### Distribution

Philippine-endemic

#### Micronecta quadristrigata

Breddin, 1905

9469CBCE-591B-57E0-A537-0740E3D3896C

https://www.gbif.org/species/4781040

#### Micronecta

Kirkaldy, 1897

DEB8B491-3078-5A71-9031-049D1884C762

##### Notes

unidentified species

#### Asthenocoris luzonensis paradisianus

Zettel & Nieser, 1999

4BBCC3A5-CAD0-5F80-A674-EC680CE72E3E

https://www.gbif.org/species/9414246

##### Distribution

Mindoro-endemic

#### Aphelocheirus (Aphelocheirus) freitagi

Zettel & Pangantihon, 2010

EC9EDCB4-23F8-5B38-8887-4A838BD98D16

https://www.gbif.org/species/9307826

##### Distribution

Mindoro-endemic

#### Anisops kuroiwae

Matsumura, 1915

61D1201D-83FE-5017-9097-8F956BFDBBCC

https://www.gbif.org/species/2020497

#### Anisops nigrolineatus

Lundblad, 1933

728576DA-67D7-5914-88FD-F46439F58856

https://www.gbif.org/species/10548908

##### Notes

new Mindoro record

#### Anisops rhomboides

Nieser & Chen, 1999

EB448517-167B-5AF0-81FC-2EA5817179BC

https://www.gbif.org/species/9030290

##### Notes

new Mindoro record

#### Anisops stali

Kirkaldy, 1897

112B0489-0C2D-544E-88AD-709C46D3B6F5

https://www.gbif.org/species/4780156

#### Nychia sappho

Kirkaldy, 1901

2386149A-82C5-54AE-9E42-609932DA68B3

https://www.gbif.org/species/5813019

#### Enithares bakeri

Brooks, 1948

BEBA996A-F495-5540-A72A-CD35FC7FC695

https://www.gbif.org/species/8823438

#### Enithares martini mindoroensis

Nieser & Zettel, 1999

F0FC9EC7-E02D-5176-A615-89B5EC5BB1F5

https://www.gbif.org/species/10363548

##### Distribution

Mindoro-endemic

#### Hydrotrephes stereoides mindoroensis

Zettel, 2003

26B234CD-3275-515F-A8B0-A35B05D4F4F0

https://www.gbif.org/species/9598999

##### Distribution

Mindoro-endemic

### Checklist of the Gerromorpha of Mindoro

#### Hebrus haddeni

Porter, 1954

F21EE14B-78B9-559F-BAF6-F498A1B2D521

https://www.gbif.org/species/9464372

##### Distribution

Philippine-endemic

#### Hebrus hoberlandti

Porter, 1959

F90E55BC-F9E7-5D43-B84A-B99F51A81120

https://www.gbif.org/species/9015705

##### Distribution

Philippine-endemic

##### Notes

unpublished records as stated by Zettel (2014)

#### Hebrus philippinus

Zettel, 2006

BB204543-76C3-5C90-9159-79A4AB951EB8

https://www.gbif.org/species/9619952

##### Distribution

Philippine-endemic

##### Notes

new Mindoro record

#### Hebrus

Curtis, 1833

9DB33643-0676-5394-8C3C-FF9A073C6BC7

##### Notes

unidentified species of the *H. harrisi* complex

#### Mesovelia cf. horvathi

Lundblad, 1933

6698FD98-9A91-54EF-83DC-451BC8207F1E

##### Notes

Possibly an undescribed species of the *M. horvarti* complex

#### Mesovelia vittigera

Horváth, 1895

151DD3A6-8182-5DCC-B701-554CB135E61D

#### Hydrometra julieni

Hungerford & Evans, 1934

7A710BD8-B155-5825-BA73-10DFF1D2C376

#### Hydrometra lineata

Eschscholtz, 1822

B0C32F18-FDAA-5143-A289-151F25598D3E

https://www.gbif.org/species/9293320

#### Hydrometra mindoroensis

Polhemus, 1976

C8A7BE0A-F220-57B0-A126-F0897CC1C110

https://www.gbif.org/species/9615221

#### Hydrometra orientalis

Lundblad, 1933

926FAD4A-DA34-507C-B16D-B7B65B019F72

https://www.gbif.org/species/9499832

#### Microvelia douglasi

Scott, 1874

D63CC2ED-F6E7-556D-A823-D83A8B200551

https://www.gbif.org/species/6130046

#### Pseudovelia cf. curvata

Hecher, 2006

B9B8BFCC-547C-5CDB-8549-F391ACB01A2F

##### Notes

New Mindoro record, varies slightly from the typical form from north Luzon

#### Halovelia bergrothi

Esaki, 1926

56FDEF47-5493-5E74-A184-38F35CFA6C4D

https://www.gbif.org/species/6454022

#### Halovelia esakii

Andersen, 1989

A46055DA-3751-55CC-8274-3E5E046E58EE

https://www.gbif.org/species/6454007

#### Haloveloides christyae

Zettel, 1998

8467593A-17B9-5C5D-8ABC-187339F9C479

https://www.gbif.org/species/8159203

##### Distribution

Philippine-endemic

#### Strongylovelia mindoroensis

Lansbury & Zettel, 1997

D23BD87F-B72E-5206-BB47-9F99070A64C9

https://www.gbif.org/species/9240789

##### Distribution

Mindoro-endemic

#### Xenobates

Esaki, 1930

5AFCEAA5-0C0A-5124-8C95-85EED3001A3A

##### Notes

unidentified species as stated by Pangantihon et al. (2016)

#### Angilia philippiensis

Drake & Hoberlandt, 1953

C5D509C1-678D-5B64-B78E-37C0107C9277

https://www.gbif.org/species/9304493

##### Distribution

Philippine-endemic

#### Rhagovelia cotabatoensis

Hungerford & Matsuda, 1961

1E1DDA0E-D94E-5B06-9911-DE84D176956A

##### Distribution

Philippine-endemic

##### Notes

unpublished records as stated by Zettel (2014)

#### Rhagovelia mindoroensis

Zettel, 1994

C141445A-AC54-553C-BB61-D57F9768139D

https://www.gbif.org/species/9348222

##### Distribution

Mindoro-endemic

#### Rhagovelia potamophila

Zettel, 1996

A15A0092-345A-5551-ABEC-EF280F783285

https://www.gbif.org/species/9281967

##### Distribution

Mindoro-endemic

#### Rhagovelia raddai

Zettel, 1994

1CBF62AB-4A24-5CFC-A370-65CF9A237E25

https://www.gbif.org/species/9396752

##### Distribution

Mindoro-endemic

#### Rhagadotarsus (Rhagodotarsus) kraepelini

Breddin, 1905

0F643250-6B3D-5D1E-AB58-5BE524F12A01

https://www.gbif.org/species/5866044

#### Aquarius philippinensis

Zettel & Ruiz, 2003

975D94D4-1543-58D5-A96D-B47CE4C0B162

https://www.gbif.org/species/9739670

##### Distribution

Philippine-endemic

#### Limnogonus hungerfordi

Andersen, 1975

F2D937F4-96D3-58CA-8D07-70072ADB823D

https://www.gbif.org/species/4773682

#### Limnogonus nitidus

(Mayr, 1865)

10D0A1D3-153A-5EA4-9EC1-C39CC388F6A7

https://www.gbif.org/species/8114430

#### Limnometra ciliata

Mayr, 1865

E4D41825-00DE-5BD7-B28B-684FBECF084F

https://www.gbif.org/species/9688002

#### Limnometra nigripennis nigripennis

Mayr, 1865

341C17B2-2341-59EB-89A8-C2475E7A31F5

https://www.gbif.org/species/9731155

##### Distribution

Philippine-endemic

#### Limnometra rossii

Hungerford & Matsuda, 1958

AF96C455-03F9-5058-9AE3-9F399D7F4A16

https://www.gbif.org/species/9790486

##### Distribution

Mindoro-endemic

#### Tenagogonus

Stål, 1853

FE873AB9-FFB9-5154-BAC5-B0FBE21C5280

#### Rheumatogonus luzonicus

(Kirkaldy, 1909)

CA7FEF25-B26C-5297-B998-6CE52BAB279F

https://www.gbif.org/species/9684453

##### Distribution

Philippine-endemic

#### Halobates calyptus

Herring, 1961

FE8E4707-152A-5E94-A81C-EB92A0C7B96A

https://www.gbif.org/species/6453976

#### Halobates maculatus

Schadow, 1922

A0F79B98-43DC-54E5-86B1-3D1A3943D62E

https://www.gbif.org/species/6453984

#### Metrocoris tenuicornis

Esaki, 1926

1A031821-6BF2-5872-8C52-426995B920B6

https://www.gbif.org/species/9764735

## Discussion

Fifty-one species of Gerromorpha and Nepomorpha are known from Mindoro, of which 29 were documented in this study. Four of them were new records to the Island, namely *Anisops nigrolineatus*, *Anisops rhomboides*, *Ochterus magnus* and *Hebrus philippinus*. Some of the remaining species/subspecies are common and widespread in the Philippines and neighbouring areas, while nine are endemic to Mindoro (see checklists above). In addition, at least three species are likely to be new to science, although we refrain from a formal description here.

As generally observed in the Philippines and adjacent areas, Veliidae (riffle bugs) and Gerridae (true water striders) are the most speciose families. With the two new records of *Anisops*, Notonectidae are also surprisingly diverse.

The biogeographic history of the Island is partly reflected by the species assemblages, especially in terms of a good number of island-endemic species. Unlike many other Philippine Islands of marine origin, Mindoro belongs to the so-called Palawan Microcontinental Block, a fragment of the Eurasian continental margin (Hall 2002). This is, however, not notably reflected in the current species composition of water bugs, since more recent dispersal and species radiation mechanisms might have had more impact on the current species distribution. Amongst the taxa treated here, only *Metrocoris tenuicornis* Esaki, 1926 (see Esaki 1926) has a distinct Palawan-Mindoro distribution range in the Philippines (although it is widely distributed in southeast Asia). An unusual pattern is observed for some closely-related species/subspecies of the *Rhagovelia papuensis* Lundblad, 1936 (see Lundblad 1936) group, of which two different subspecies of *Rhagovelia kawakamii* (Matsumura, 1913) (see Matsumura 1913) are distributed in either Borneo and Palawan or Taiwan and Luzon, respectively, but missing on Mindoro Island, where it is replaced by the closely-related *R. mindoroensis* (Zettel et al. 2020).

Despite their close vicinity to Luzon, the Islands remained always disconnected during the Quaternary (Hall 2002). Nevertheless, Mindoro and the eastern Philippine Islands share a large proportion of water bugs.

Amongst the species of the checklist that were unambiguously identified, 20% are endemic to Mindoro, another 28% Philippine-endemic, making almost half of all species endemic to the country. The island-endemism rate is slightly lower than in Palawan, with one-third endemism amongst aquatic and semi-aquatic bugs (Freitag and Zettel 2012). This is likely due to the closer vicinity of Mindoro to other intra-Philippine biogeographic regions - notably Luzon - enabling easier dispersal across sea barriers.

In this study, special emphasis was given to the collection in lotic systems, which might have led to an under-representation of typical pond- and lake-dwelling species. Nevertheless, stream-associated lentic microhabitats, such as side pools and residual pools, were sampled in most collecting sites. Representatives of the genera *Anisops*, *Enithares*, *Hydrometra*, *Micronecta*, *Microvelia* and *Ranatra* are typically found there, but also *Rhagadotarsus kraepelini* (which is usually a pond or lake dweller) (Freitag and Pangantihon 2010, current study).

Worth noting is that hygropetric microhabitats are an important habitat for several, partly rare and endemic species, foremost of these being *Hebrus philippinus*, *Hydrometra mindoroensis*, *Mesovelia vittigera* and *Ochterus* spp. Such habitas are particularly threatened by deforestation and land-use changes since they are prone to drying up when not continuously fed with water from the forested areas or when they are fully exposed to direct sunlight.

Fast flowing or even torrent waters, on the other hand, are typically inhabited by species of *Aphelocheirus*, *Asthenocoris* and *Hydrotrephes*, as well as *Rhagovelia raddai* amongst the taxa treated here.

A few nepomorphan taxa are particularly attracted to light. *Micronecta* sp. and *Anisops kuroiwae* were only retrieved by black light traps in this study. Emergence traps rarely yield aquatic and riparian Heteroptera. We caught only very few specimens of *Ochterus polhemusi* and *Hebrus* sp. in such traps.

## Supplementary Material

XML Treatment for Ranatra sp.

XML Treatment for Ochterus magnus

XML Treatment for Ochterus polhemusi

XML Treatment for Micronecta sp.

XML Treatment for Asthenocoris luzonensis paradisianus

XML Treatment for Aphelocheirus freitagi

XML Treatment for Anisops kuroiwae

XML Treatment for Anisops nigrolineatus

XML Treatment for Anisops rhomboides

XML Treatment for Enithares martini mindoroensis

XML Treatment for Hydrotrephes stereoides mindoroensis

XML Treatment for Hebrus philippinus

XML Treatment for Hebrus sp.

XML Treatment for Mesovelia horvathi

XML Treatment for Mesovelia vittigera

XML Treatment for Hydrometra lineata

XML Treatment for Hydrometra mindoroensis

XML Treatment for Microvelia douglasi

XML Treatment for Pseudovelia curvata

XML Treatment for Strongylovelia mindoroensis

XML Treatment for Rhagovelia mindoroensis

XML Treatment for Rhagovelia raddai

XML Treatment for Rhagovelia potamophila

XML Treatment for Rhagadotarsus (Rhagadotarsus) kraepelini

XML Treatment for Limnogonus nitidus

XML Treatment for Limnometra nigripennis nigripennis

XML Treatment for Tenagogonus sp.

XML Treatment for Rheumatogonus luzonicus

XML Treatment for Metrocoris tenuicornis

XML Treatment for Ranatra

XML Treatment for Ochterus polhemusi

XML Treatment for Ochterus magnus

XML Treatment for Ochterus marginatus insularis

XML Treatment for Ochterus philippinensis

XML Treatment for Micronecta quadristrigata

XML Treatment for Micronecta

XML Treatment for Asthenocoris luzonensis paradisianus

XML Treatment for Aphelocheirus (Aphelocheirus) freitagi

XML Treatment for Anisops kuroiwae

XML Treatment for Anisops nigrolineatus

XML Treatment for Anisops rhomboides

XML Treatment for Anisops stali

XML Treatment for Nychia sappho

XML Treatment for Enithares bakeri

XML Treatment for Enithares martini mindoroensis

XML Treatment for Hydrotrephes stereoides mindoroensis

XML Treatment for Hebrus haddeni

XML Treatment for Hebrus hoberlandti

XML Treatment for Hebrus philippinus

XML Treatment for Hebrus

XML Treatment for Mesovelia cf. horvathi

XML Treatment for Mesovelia vittigera

XML Treatment for Hydrometra julieni

XML Treatment for Hydrometra lineata

XML Treatment for Hydrometra mindoroensis

XML Treatment for Hydrometra orientalis

XML Treatment for Microvelia douglasi

XML Treatment for Pseudovelia cf. curvata

XML Treatment for Halovelia bergrothi

XML Treatment for Halovelia esakii

XML Treatment for Haloveloides christyae

XML Treatment for Strongylovelia mindoroensis

XML Treatment for Xenobates

XML Treatment for Angilia philippiensis

XML Treatment for Rhagovelia cotabatoensis

XML Treatment for Rhagovelia mindoroensis

XML Treatment for Rhagovelia potamophila

XML Treatment for Rhagovelia raddai

XML Treatment for Rhagadotarsus (Rhagodotarsus) kraepelini

XML Treatment for Aquarius philippinensis

XML Treatment for Limnogonus hungerfordi

XML Treatment for Limnogonus nitidus

XML Treatment for Limnometra ciliata

XML Treatment for Limnometra nigripennis nigripennis

XML Treatment for Limnometra rossii

XML Treatment for Tenagogonus

XML Treatment for Rheumatogonus luzonicus

XML Treatment for Halobates calyptus

XML Treatment for Halobates maculatus

XML Treatment for Metrocoris tenuicornis

## Figures and Tables

**Table 1.** Acronym codes of sampling sites in the Province of Oriental Mindoro as used in the result section.

**Code**	**Municipality/ Barangay**	**River/Creek/ Tributary**	**Description**	**Latitude, Longitude**	**Elevation** **(m asl)**
301	Puerto Galera	Tagbinai Munti River	hill creek in coconut plantation	13°29'00''N, 120°57'12''E	10
302	Puerto Galera	downstream of Tamaraw Falls	creek in secondary vegetation	13°27'03"N, 120°59'27"E	80
303	Puerto Galera, Calsapa	Tukunan River (“Hidden Paradise”) (Fig. 1C)	medium-sized river in secondary vegetation	13°26'00''N, 120°58'23''E	80
304	Puerto Galera	downstream of Aninuan Falls	creek in secondary vegetation	13°29'10''N, 120°54'18''E	10
305	Puerto Galera	Tagbinai Malaki River (Fig. 1B)	small river in secondary vegetation	13°28'57''N, 120°57'34''E	30
310	Baco, Dulangan	Lantuyan River	torrent mountain river in secondary vegetation	13°16'08''N, 121°04'56''E	55
312	Baco, Dulangan	Lantuyan River	torrent tributary of Lantuyan River in secondary forest	13°18'02"N, 121°02'44"E	400
353	Baco	Baco, Rural Road Side	residual pools of small intermittent river in secondary vegetation	13°21'49''N, 121°05'30''E	26
356	Baco, Dulangan	lower Dulangan River (Fig. 1D)	torrent river in rural open land	13°21'22''N, 121°07'10''E	8
396	Puerto Galera, Poblacion	lowland Creek (Fig. 1A)	small lowland creek in secondary vegetation	13°30'07"N, 120°56'46"E	2
BR2	Roxas, San Mariano	middle Baroc River	disturbed warm water river in farmland	12°37'40"N, 121°26'29"E	10
BR3	Roxas, Wasig	lower Baroc River (Fig. 2A)	disturbed warm water river in farmland	12°35'51''N, 121°28'11''E	2
HBC	Roxas, San Vicente	Quirao Buhay Creek	creek in secondary vegetation	12°36'10''N, 121°23'00''E	142
HBT	Roxas, San Vicente	Tagugoy Creek	small Quirao Buhay tributary in secondary forest	12°36'30''N, 121°22'38''E	200
HTC	Roxas, San Vicente	Tinggiwang Creek	creek in secondary forest	12°35'48''N, 121°22'01''E	162
HQC	Roxas, San Vicente	Quirao na Balete Creek	mountain creek fringed by secondary forest	12°35'38''N, 121°23'34''E	230
HR1	Roxas, San Vicente, Quirao	Hinundungan River	slightly disturbed lowland river in rural extensive farmland and secondary vegetation	12°36'23''N, 121°23'29''E	118
HR2	Roxas, San Vicente	Hinundungan River down-stream of Hinagdanan Falls (Fig. 2B)	clean mountain river in secondary forest	12°35'23''N, 121°21'52''E	200
HR3	Roxas, San Vicente	Hinundungan River up-stream of Hinagdanan Falls	clean mountain river in secondary forest	12°35'10''N, 121°21'36''E	280
TAC	Roxas, San Vicente	Sapang Alupa	torrent creek in old secondary forest	12°37'48''N, 121°20'52''E	340
TDR1	Roxas, San Vicente, Taugad Diit	lower Taugad Daka River	clean mountain river in extensive farmland and secondary forest	12°37'33''N, 121°21'18''E	180
TDR4	Roxas, San Vicente	upper Taugad Daka River (Fig. 2C)	clean mountain creek in secondary forest	12°38'00''N, 121°19'15''E	700
THC	Roxas, San Vicente	Hiyong Creek	perennial creek in extensive farmland and secondary vegetation	12°37'27''N, 121°22'48''E	147
THF	Roxas, San Vicente	Hiyong Fall	small fall of perennial creek in extensive farmland and secondary vegetation	12°37'32''N, 121°22'47''E	150
TIR1	Roxas, San Vicente, Taugad Diit	lower Taugad Diit River	slightly disturbed river in extensive farmland and secondary vegetation	12°37'32"N, 121°22'17"E	180
TR1	Roxas, San Vicente Proper	Taugad River	slightly disturbed lowland river in extensive farmland and secondary vegetation	12°37'07''N, 121°23'37"E	95
TR2	Roxas, San Vicente	upper Taugad River	mountain river in secondary vegetation and forest	12°37'18''N, 121°22'58''E	140
TUC	Roxas, San Vicente	“unnamed” creek (Fig. 2D)	small intermittent creek in secondary forest	12°37'38''N, 121°22'38''E	154
TWC	Roxas, San Vicente Proper	“community water source” creek”	perennial mountain creek in secondary forest	12°37'01"N, 121°23'18"E	150

**Table 2.** Microhabitat codes for samples as used in the result section.

**Microhabitat code**	**Description**
a	littoral sand/gravel in running sections of the stream/river
b	mud/sand/fine gravel in littoral pool sections with stagnant or very slow-moving water connected to the stream/river (Fig. 3B)
c	stream bottom gravel in running sections of the stream/river (Fig. 3C)
d	leaf packs in running and riffle sections of the stream/river (Fig. 3D)
e	leaf litter/CPOM in isolated side pools or residual pool (separated from stream)
f	submerged wood in running and riffle sections of the stream/river
g	solid rock surfaces in riffle and running sections of the stream/river (Fig. 3F)
h	root packs/grass bunches in running sections of the stream/river (Fig. 3G)
j	hygropetric rocks
k	CPOM/leaf litter in small side rivulets connected to the mainstream (Fig. 3E)
m	gravel/sand in shallow, sun-exposed side pools or residual pool (separated from stream)
t	littoral gravel/sand/mud deposits of side pools or residual pool (separated from stream) (Fig. 3A)
u	water plants inside pools or residual pool (separated from stream)
y	water surface (neustic) of calm water sections (pool)
z	water surface (neustic) of running and riffle water sections (Fig. 3H)

## References

[B5978683] Andersen N. M., Yang C. M., Zettel H. (2002). Notes on the Microveliinae of Singapore and Peninsular Malaysia with the description of the two new species of *Microvelia* WESTWOOD (Hemiptera-Heteroptera: Veliidae. The Raffles Bulletin of Zoology.

[B5981517] Andersen N. M., Weir T. A. (2003). The genus *Microvelia* Westwood in Australia (Hemiptera : Heteroptera : Veliidae). Invertebrate Taxonomy.

[B5982191] Breddin G. (1905). Rhynchota
Heteroptera aus Java. Mitteillungen aus dem Naturhistorischen Museum.

[B5978692] Chandra K., Jehamalar E. E. (2011). New records of Gerromorpha, Leptopodomorpha and Nepomorpha (Heteroptera, Insecta) from Madhya Pradesh, India. Biodiversity Journal.

[B5980308] Cheng L., Yang C. M., Andersen N. M. (2001). Guide to the aquatic Heteroptera of Singapore and Peninsular Malaysia. I. Gerridae and Hermatobatidae. Raffles Bulletin of Zoology.

[B5978710] Chen P. P., Nieser N. (1993). A taxonomic revision of the Oriental water strider genus *Metrocoris* Mayr (Hemiptera, Gerridae) Part I and II. Steenstrupia.

[B5978719] Chen P. P., Nieser N. (2002). Taxonomic characters of the male endosomal structure in the genus *Rheumatogonus* Kirkaldy (Hemiptera: Gerridae), with descriptions of four new species from Borneo and Sri Lanka. Zoologische Mededelingen.

[B5978737] Chen P. P., Nieser N., Zettel H. (2005). The aquatic and semi-aquatic bugs (Heteroptera: Nepomorpha & Gerromorpha) of Malesia..

[B5982146] Dallas W. S. (1850). Notice of some Hemiptera from Boutan in the collection of the Hon. East India Company. Transactions of the Royal Entomological Society of London..

[B5980461] Damgaard J., Moreira F. F., Weir T. A., Zettel H. (2014). Molecular phylogeny of the pond skaters (Gerrinae), discussion of the fossil record and a checklist of species assigned to the subfamily (Hemiptera: Heteroptera: Gerridae). Insect Systematics & Evolution.

[B5982045] Esaki T. (1926). The water-striders of the subfamily Halobatinae in the Hungarian National Museum. Annales Historico-naturales Musei Nationalis Hungarici.

[B5982200] Eschscholtz J. F. (1822). Entomographien.

[B5982164] Fabricius J. C. (1775). Systema Entomologiae, Sistens Insectorvm Classes, Ordines, Genera, Species, Adiectis Synonymis, Locis, Descriptonibus, Observationibus. Flensburgi et Lipsiae: Kort.

[B5978745] Freitag H. (2004). Adaptations of an emergence trap for use in tropical streams. International Revue of Hydrobiology.

[B5979134] Freitag Hendrik, Pangantihon Clister V (2010). Aquatic Coleoptera and Heteroptera of the Lake Naujan National Park, Oriental Mindoro, the Philippines. Philippine Scientist.

[B5979143] Freitag Hendrik, Zettel Herbert (2012). Aquatic Heteroptera of the Lake Manguao catchment, Palawan and new rank of *Rhagovelia
kawakamii
hoberlandti* Hungerford & Matsuda, 1961. Philippine Journal of Systematic Biology.

[B5978754] Freitag H. (2013). *Ancyronyx* Erichson, 1847 (Coleoptera, Elmidae) from Mindoro, Philippines, with description of the larvae and two new species using DNA sequences for the assignment of the developmental stages. ZooKeys.

[B5982036] Gapud V. P., San Valentin H. O. (1977). Ochteridae (Hemiptera) of the Philippines. Kalikasan Philippine Journal of Biology.

[B5979152] Gapud V. P. (1981). Contribution to the taxonomy of the genus *Ochterus* Latreille (Hemiptera: Ochteridae). Kalikasan Philippine Journal of Biology.

[B5979164] Gapud V. P. (1986). Philippine water bugs. Guide to Philippine Flora and Fauna.

[B5979173] Gapud V. P. (1995). A new species of *Ochterus* Latreille (Hemiptera: Ochteridae) from the Philippines. Asia Life Sciences.

[B5979191] Gapud V. P., Zettel H. (1999). The Philippine Water Bug Inventory Project (PWBIP) and a bibliography for Philippine Nepomorpha, Gerromorpha, and Leptopodomorpha (Insecta: Heteroptera. Annalen Des Naturhistorischen Museums in Wien.

[B5979182] Gapud V. P. (2002). Two new Philippine *Ochterus* Latreille (Insecta: Heteroptera: Ochteridae) and checklist of Philippine species. Annalen Des Naturhistorischen Museums in Wien.

[B5979200] Gapud V. P., Zettel H., Yang C. M. (2003). The Hydrometridae (Insecta: Heteroptera) of the Philippine Islands. Annalen des Naturhistorischen Museums in Wien.

[B5979209] Garces J. M., Bauernfeind E., Freitag H. (2018). *Sparsorythus
sescarorum*, new species from Mindoro, Philippines (Ephemeroptera, Tricorythidae). ZooKeys.

[B5979218] Hall R. (2002). Cenozoic geological and plate tectonic evolution of SE Asia and the SW Pacific: Computer-based reconstructions, model and animations. Journal of Asian Earth Sciences.

[B5979245] Hecher C. (2006). Review of the genus *Pseudovelia* (Heteroptera, Veliidae) on the Philippines: Part II: Greater Luzon. Denisia.

[B5982080] Horváth G. (1895). Hemiptères nouveaux d'Europe et des pays limitrophes. Revue d'Entomologie.

[B5982182] Hungerford H. B., Matsuda R. (1958). The *Tenagogonus*-*Limnometra* complex of the Gerridae. The University of Kansas Science Bulletin.

[B5979254] Jehamalar E. E., Chandra K., Polhemus D. (2019). Review of the *Mesovelia
horvathi* species complex (Hemiptera: Gerromorpha: Mesoveliidae), with the description of seven new species from India. Zootaxa.

[B5982226] Kirkaldy G. W. (1909). Hemiptera, old and new, No. 2. The Canadian Entomologist.

[B5979263] Komarek A., Freitag H. (2014). Revision of *Anacaena* Thomson, 1859. XI. Republic of the Philippines (Coleoptera: Hydrophilidae). Koleopterologische Rundschau.

[B5979272] Komarek A., Freitag H. (2020). Taxonomic revision of *Agraphydrus* Régimbart, 1903 IV. Philippines (Coleoptera: Hydrophilidae: Acidocerinae).. Koleopterologische Rundschau.

[B5979328] Kormilev N. A. (1971). Ochteridae from the Oriental and Australian regions (Hemiptera, Heteroptera). Pacific Insects.

[B5982155] Lansbury I. (1972). A review of the Oriental species of *Ranatra* Fabricius (Hemiptera-Heteroptera: Nepidae). Transactions of the Royal Entomological Society of London..

[B5979376] Lansbury I., Zettel H. (1997). New species and subspecies of the genus *Strongylovelia* ESAKI (Insecta: Heteroptera: Veliidae) from Borneo and the Philippines. Annalen des Naturhistorischen Museums in Wien.

[B5982027] Lundblad O. (1933). Zur Kenntnis der aquatilen und semi-aquatilen Hemipteren von Sumatra, Java und Bali. Archiv für Hydrobiologie. Supplement..

[B5982054] Lundblad O. (1936). Die altweltlichen Arten der Veliidengattungen *Rhagovelia* und *Tetraripis*. Arkiv för Zoologi..

[B5979392] Lundblad O. (1937). Einige neue oder wenig bekannte ostasiatische *Rhagovelia*-Arten. Entomologisk Tidskrift.

[B5982063] Matsumura S. (1913). Thousands Insects of Japan. Additamenta.

[B5982089] Matsumura S. (1915). Übersicht der Wasser-Hemipteren von Japan und Formosa. Entomological Magazine, Kyoto.

[B5982173] Mayr G. L. (1865). Diagnosen Neuer Hemipteren II. Verhandlungen der zoologisch-botanischen Gesellschaft in Wien.

[B5979401] Mey W., Freitag H. (2013). Trichoptera of Mindoro, the Philippines I. New species and records from the Baroc River Catchment, Roxas, Oriental Mindoro (Insecta, Trichoptera). Esperiana Band.

[B5979410] Nieser N. (1999). Introduction to the Micronectidae (Nepomorpha) of Thailand. Amemboa.

[B5979419] Nieser N., Chen P. P. (1999). Sixteen new species of Nepomorpha (Heteroptera) mainly from Sulawesi (Indonesia). Tijdschrift voor Entomologie.

[B5979445] Nieser N., Zettel H. (1999). The *Enithares* Spinola, 1837 (Insecta: Heteroptera: Notonectidae) of the Philippines, with descriptions of two new taxa. Annalen des Naturhistorischen Museums in Wien.

[B5979454] Nieser N., Chen P. P. (2003). Four new taxa of *Micronecta* from the Philippines (Insecta: Heteroptera: Micronectidae. Annalen des Naturhistorischen Museums in Wien.

[B6298271] Nieser N. (2004). Guide to aquatic Heteroptera of Singapore and peninsular Malaysia III. Pleidae and Notonectidae. Raffles Bulletin of Zoology.

[B5979463] Ong P. S., Afuang L. E., Rosell-Ambal R. C. (2002). Philippine biodiversity conservation priorities: A second iteration of the National Biodiversity Strategy and Action Plan.

[B5979472] Pangantihon C. V., Freitag H. (2016). New Records of Philippine True Bugs (Hemiptera: Heteroptera) with coastal and marine habitat associations and a checklist of Philippine species. Philippine Scientist.

[B5979496] Pelingen A. L., Freitag H. (2020). Description of *Neoperla
mindoroensis* sp. nov., the first record of a stonefly from Mindoro, Philippines (Plecoptera, Perlidae), and identification of its life stages using COI barcodes. Zookeys.

[B5982071] Polhemus J. T., Reisen W. K. (1976). Aquatic Hemiptera of the Philippines. Kalikasan Philippine Journal of Biology.

[B5979532] Polhemus J. T., Karunaratne P. B. (1993). A review of the genus *Rhagadotarsus*, with descriptions of three new species (Heteroptera: Gerridae). Raffles Bulletin of Zoology.

[B5979523] Polhemus J. T., Lansbury I. (1997). Revision of the genus *Hydrometra* LATREILLE in Australia, Melanesia, and the southwest Pacific (Heteroptera: Hydrometridae). Bishop Museum Occasional Papers.

[B5981019] Scott J. (1874). On a collection of Hemiptera
Heteroptera from Japan. Descriptions of various new genera and species. Annals and Magazine of Natural History, Series 4..

[B5979615] Vidal A. R., Go K. C.T.S., Freitag H. (2017). Hydraenidae (Insecta: Coleoptera) of Mindoro, Philippines. I: *Hydraena* Kugelann, 1794 of the Baroc River basin, Roxas, Oriental Mindoro with description of three new species. Aquatic Insects.

[B5979633] Yang C. M., Murphy D. (2011). Guide to the Aquatic Heteroptera of Singapore and Peninsular Malaysia. 6. Mesoveliidae, with description of a new species from Singapore. The Raffles Bulletin of Zoology.

[B5979656] Zettel H. (1994). Zwei neue *Rhagovelia*-Arten aus Mindoro, Philippinen (Heteroptera, Vellidae). Entomofauna.

[B5980894] Zettel H. (1996). Revision der philippinischen Arten der Gattung *Rhagovelia*, 3. Teil (Heteroptera: Veliidae). Entomological Problems.

[B5979750] Zettel H., Nieser N., Polhemus D. A. (1999). The Naucoridae (Insecta:Heteroptera) of the Philippine Islands. Annalen des Naturhistorischen Museums in Wien.

[B5979683] Zettel H. (2003). Additional notes on the Aphelocheiridae, Naucoridae, and Notonectidae (Insecta: Heteroptera: Nepomorpha) of the Philippine Islands. Annalen des Naturhistorischen Museums in Wien.

[B5980201] Zettel H. (2003). The Helotrephidae (Insecta: Heteroptera) of the Philippine Islands. Annalen des Naturhistorischen Museums in Wien.

[B5980916] Zettel H. (2003). New species, subspecies, and records of *Strongylovelia* ESAKI, 1924 (Insecta: Heteroptera: Veliidae) from the Philippines. Annalen des Naturhistorischen Museums in Wien.

[B5979692] Zettel H. (2004). Neue Arten der Gattung *Hebrus* CURTIS 1833 (Heteroptera: Hebridae) aus Südostasien. Linzer biologische Beiträge.

[B5980383] Zettel H. (2004). Neue Wasserläufer (Insecta: Heteroptera: Gerridae) von den Philippinen. Annalen des Naturhistorischen Museums in Wien.

[B5979701] Zettel H. (2006). Neue Arten der Gattung *Hebrus* CURTIS 1833 (Heteroptera: Hebridae) aus Südostasien - 2. Teil.. Linzer biologische Beiträge.

[B5979759] Zettel H., Pangantihon C. V. (2010). Aphelocheirus
(s.str.)
freitagi nov. sp. from Mindoro Island and additional notes on Philippine Aphelocheiridae (Heteroptera). Linzer biologische Beiträge.

[B5979768] Zettel H., Lane D. J.W., Pangantihon C. V., Freitag H. (2012). Notes on Notonectidae (Hemiptera: Heteroptera) from southeastern Asia, mostly from Brunei and the Philippines. Acto Entomologica Musei Nationalis Pragae.

[B5979710] Zettel H. (2014). Annotated catalogue of semi-aquatic bugs (Hemiptera: Heteroptera: Gerromorpha) of Luzon Island, the Philippines, with descriptions of new species. Zeitschrift der Arbeitsgemeinschaft Österreichischer Entomologen.

[B5979777] Zettel H., Phauk S., Kheam S., Freitag H. (2017). Checklist of the aquatic Hemiptera (Heteroptera: Gerromorpha and Nepomorpha) of Cambodia, with descriptions of new species of *Microvelia* Westwood, 1834 and *Ranatra* Fabricius, 1790. Aquatic Insects.

[B5982741] Zettel H., Laciny A., Freitag H. (2020). Review of the genus *Rhagovelia* (Insecta: Heteroptera: Veliidae) in the Palawan biogeographic region, the Philippines. Raffles Bulletin of Zoology.

